# Effects of water flow and ocean acidification on oxygen and pH gradients in coral boundary layer

**DOI:** 10.1038/s41598-024-63210-9

**Published:** 2024-06-04

**Authors:** Catarina P. P. Martins, Maren Ziegler, Patrick Schubert, Thomas Wilke, Marlene Wall

**Affiliations:** 1 Department of Animal Ecology & Systematics, Justus Liebig University Giessen, 35392 Giessen, Germany; 2 GEOMAR Helmholtz Centre for Ocean Research Kiel, 24148 Kiel, Germany

**Keywords:** Climate-change ecology, Marine biology, Ecophysiology, Tropical ecology

## Abstract

Reef-building corals live in highly hydrodynamic environments, where water flow largely controls the complex chemical microenvironments surrounding them—the concentration boundary layer (CBL). The CBL may be key to alleviate ocean acidification (OA) effects on coral colonies by partially isolating them. However, OA effects on coral CBL remain poorly understood, particularly under different flow velocities. Here, we investigated these effects on the reef-building corals *Acropora cytherea*, *Pocillopora verrucosa*, and *Porites cylindrica*. We preconditioned corals to a control (pH 8.0) and OA (pH 7.8) treatment for four months and tested how low flow (2 cm s^−1^) and moderate flow (6 cm s^−1^) affected O_2_ and H^+^ CBL traits (thickness, surface concentrations, and flux) inside a unidirectional-flow chamber. We found that CBL traits differed between species and flow velocities. Under OA, traits remained generally stable across flows, except surface pH. In all species, the H^+^ CBL was thin and led to lower surface pH. Still, low flow thickened H^+^ CBLs and increased light elevation of surface pH. In general, our findings reveal a weak to null OA modulation of the CBL. Moreover, the OA-buffering capacity by the H^+^ CBL may be limited in coral species, though low flow could enhance CBL sheltering.

## Introduction

Coral reefs are highly hydrodynamic habitats, with spatially variable hydrodynamic regimes depending on reef zones and structures (e.g., uniform calm flow in lagoons vs high-energy wave-driven flow on reef crests)^1^. Importantly, water flow also varies temporally in reefs, where flow velocities may range from 0 to 30 cm s^−1^ within single locations^2^ and some sites may present low flow lasting only a few hours^3^.

For reef-building corals, water flow is a key environmental factor due to its influence on the coral concentration boundary layer (CBL)^4^. It represents a thin layer of seawater bordering the coral surface where concentration gradients of dissolved compounds (e.g., gases^5^ and nutrients^6^) are formed between the coral and bulk seawater^7^. These gradients shape key physiological processes, such as photosynthesis, and their properties are largely determined by water flow conditions^8^. For instance, under reduced water flow, the O_2_ CBL becomes thicker and O_2_ flux is decreased^9,10^. Besides flow conditions, the characteristics of the CBL are also dependent on coral surface topography, polyp behaviour and traits, and cilia activity^11–13^, with the steepness of gradients being further modulated by the metabolic activity of the organism itself^7^. The CBL is thus a very dynamic chemical microenvironment at the coral surface that acts as a partial chemical barrier between the coral and its environment.

Under ocean acidification (OA)—a process characterised by decreased pH and altered carbonate chemistry, with negative effects on coral metabolism^14^—the CBL may be key in partially isolating coral colonies from surrounding acidified seawater. For instance, massive coral species have shown similar surface pH under control and OA after 30-min and 5-day exposure to OA conditions^15,16^. This ability to maintain stable surface pH, despite the lower pH of the surrounding seawater, occurred while maintaining stable O_2_ CBL flux. Therefore, pH in the CBL under OA may be linked to O_2_ CBL traits. However, whether this ability to increase the elevation of surface pH under OA is also present in branching coral species remains unclear^17^. Furthermore, CBL traits could potentially be affected by OA due to changes in coral microtopography and surface cilia activity. For instance, OA has been shown to affect cilia in marine organisms^18,19^, and OA effects on coral skeletons^20^ could alter the topography of the tissue surface, modifying the flow patterns at the coral-seawater interface.

The effects of OA on CBL traits may also differ with exposure time and display complex interactions with water flow. During 30-min exposure to OA, O_2_ CBL thickness of *Favites* sp. was stable under low flow (below 2 cm s^−1^) but increased under moderate flow (5 cm s^−1^), while elevation of surface pH was larger under low flow^16^. Moreover, after over four months under OA, surface pH in light was similar to seawater pH in *Acropora yongei*, but in *Plesiastrea versipora* it was elevated above seawater level, with a greater magnitude under low than moderate flow (2.5 and 8 cm s^−1^, respectively)^17^.

Although long-term OA effects on the coral CBL remain poorly understood, differences in CBL traits may help explain the range of sensitivities to OA among reef-building corals^21^. In addition, the magnitude of daily pH fluctuation at the coral surface may further determine responses to OA. However, while coral colonies with higher surface pH in light generally also have lower surface pH in darkness^22^, CBL traits under OA are rarely documented in darkness. Finally, despite recent studies, knowledge of how water flow may modulate coral responses to OA at the scale of the CBL remains limited. Under future OA scenarios, seawater pH will decrease, and together with amplified fluctuation^23^ shift the natural variability of pH to lower values. Reef hydrodynamics will also be affected by climate change^24^, as well as sea level rise and increased storm activity^25^. Although high resolution projections of reef flow patterns are still limited^24^, individual locations may continue to experience flow ranging from high to near-zero velocities^26^. Thus, a comprehensive assessment of the interactive effects of water flow and long-term OA on O_2_ and pH gradients in the CBL of reef-building coral species is needed to better understand their responses to future climate change.

The general aim of this study was therefore to characterise the effects of water flow and OA on the CBL of three major reef-building coral species, *Acropora cytherea* (Dana, 1846), *Pocillopora verrucosa* (Ellis & Solander, 1786), and *Porites cylindrica* Dana, 1846, during light and dark conditions. Specifically, we preconditioned corals to either OA (pH 7.8) or control (pH 8.0) treatments for four months and tested how low flow (2 cm s^−1^) and moderate flow (6 cm s^−1^) affected I) thickness of O_2_ and pH gradients in the coral CBL, II) O_2_ and pH values at the coral surface, and III) O_2_ flux across the CBL, inside a unidirectional-flow chamber. Responses to OA in H^+^ CBL traits were assessed in *A. cytherea* only. Taken together, this will help disentangle the complex interacting effects of water flow and OA on coral physiology and better understand the role of the coral CBL in coral susceptibility to OA.

## Materials and methods

### Experimental design

Three scleractinian coral species, *A. cytherea*, *P. verrucosa*, and *P. cylindrica*, were preconditioned to ambient pH and OA conditions for four months, and the OA effects on the coral CBL were studied under low flow and moderate flow. Coral colonies (see Supplementary Table S1 for origin and CITES numbers) were maintained at the ‘Ocean2100’ long-term coral experimental facility (7000 L closed recirculating system of artificial seawater, Supplementary Table S2) at Justus Liebig University Giessen, Germany, for at least six months before the experiment. Conditions in the long-term culturing tanks (256 L) were 11:13 h light:dark photoperiod, with a light intensity of 230 µmol photons m^−2^ s^−1^, temperature of 26.0 ± 0.5 °C (mean ± 1 SD), flow velocity of 5–10 cm s^−1^, and daily feeding of a mix of frozen *Artemia* sp., *Mysis* sp., and copepods. For the experiment, three colonies per species were cut into six fragments (using a small angle grinder, Dremel Multitool 3000-15, The Netherlands). Fragments were attached to tiles with two-component glue (CoraFix SuperFast, Grotech, Germany) and transferred to the experimental setup. Corals were acclimated to the experimental setup for five weeks before the start of the experiment. Experimental pH treatments consisted of two levels, with the control treatment (ambient pH) mimicking present-day atmospheric *p*CO_2_ concentration in some reefs (~ 500 µatm *p*CO_2_)^27^, and the OA treatment with values projected in the long term (2081–2100) for surface ocean pH in coral reefs^28^ under SSP2-4.5 (0.20 pH units lower relative to 1961–1990)^29,30^. The experiment was conducted from 15 October 2019 to 26 March 2020. The coral response in O_2_ and H^+^ CBL traits was measured after 15–18 weeks (107–126 days) in the experimental pH treatments, taking almost three weeks to complete all microsensor measurements, during which whole-colony health was visually unchanged (i.e., no bleaching, discolourations, necrosis). Microsensor profiles were measured in light and darkness, and under bulk mean flow velocities of 2 cm s^−1^ (low flow) and 6 cm s^−1^ (moderate flow) (n = 9 profiles per species per profile condition per treatment), mimicking natural reef velocities (e.g.^2^). Details of the microsensor measurements are outlined below.

### Experimental setup and treatment conditions

The preconditioning to the two experimental pH treatments was conducted in six 120 L tanks (three tanks per treatment, nine fragments per species per treatment). Each experimental tank housed one fragment per colony (total of three fragments per species in each tank) with 15 cm spacing between them in the direction of flow. In addition, experimental tanks contained other scleractinian and octocorals with the same number of individuals per tank. The experimental tanks were supplied with water from the 7000 L closed recirculating system (calcium: 400 ± 6 mg L^−1^; phosphate: < 0.02 mg L^−1^; nitrate: < 0.02 mg L^−1^; nitrite: < 0.01 mg L^−1^) with a water inflow rate of 20–40 L h^−1^ (corresponding to a 100% tank volume turnover every 3–6 h). In addition, the large water system received weekly water changes of ~ 10% of the water volume. Temperature was maintained at 26 °C through a feedback-controlled heater (300 W, 548, Schego, Germany). Water flow was generated with two circulating pumps (ES-28, Aqualight, Germany) and one wave generator (6208, Tunze, Germany), and consisted of a standing wave with an amplitude of 5 mm and a flow velocity of 6 cm s^−1^ (OTT MF pro, OTT Hydromet GmbH, Germany), which is a common velocity across reef zones throughout the year (e.g.^2^). Flow velocity was measured in all experimental tanks at the position of the coral fragments before starting the preconditioning phase and at the end of the experiment (i.e., after all microsensor measurements were completed). Salinity in the tanks was monitored daily using a conductivity sensor (TetraCon 925, WTW, Germany) and maintained at 35. Light was provided by two T5 bulbs (54 W, Aqua-Science, Germany) producing a light intensity of 176 ± 31 µmol photons m^−2^ s^−1^ in a 11:13 h light:dark photoperiod. Corals received 2.7 mg L^−1^ of frozen copepods every two days and dissolved nutrients via the connected water system.

Seawater pH was constantly monitored using a digital controller (Profilux 3, GHL, Germany) attached to pH electrodes in each tank (GHL, Germany), which were calibrated using NBS buffers. Values of pH_NBS_ were converted to the total scale (pH_T_) and are expressed on this scale throughout the text. OA treatment conditions were generated individually via pH-controlled CO_2_ dosing (bubbling) using solenoid valves, which controlled the release of CO_2_ into each treatment tank, and pumping was done through one of the circulating pumps to aid CO_2_ dissolution and dispersion in seawater. pH was gradually decreased in OA treatment tanks and was lowered 0.01–0.02 units every day over two weeks until target values were reached. Target OA conditions were maintained for a total of 16 weeks (113 days), including diel oscillation of pH mimicking naturally occurring variability^27,31^. Total alkalinity (TA) was measured in each experimental tank by open-cell potentiometric titration using a titrator (TitroLine 7000, SI Analytics, Germany) equipped with a glass pH-combination electrode (A 162 2M-DIN-ID, SI Analytics, Germany). Measurements were made following SOP3b^32^ on 50 g samples with 0.1N HCl (Titrisol, Merk, Germany) in 35 g L^−1^ NaCl and corrected using certified reference materials (Batch 183, 194; A.G. Dickson laboratory, Scripps Institution of Oceanography, UCSD, USA)^33^. Measurements were performed every 2–4 days during the first two weeks of the experiment and then every 1–2 weeks. TA was calculated using a modified Gran approach. Alkalinity was also monitored daily and maintained with two automatic in-house constructed calcium reactors (pH 6.2–6.4, coral rubble) and dosing of NaHCO_3_ in a common reservoir tank. The calcium reactor was feedback controlled by an alkalinity controller (Alkatronic, Focustronic, Hong Kong) based on three-hourly automatic titrations.

Seawater carbonate chemistry was calculated from days with TA measurements using pH and temperature values of a whole day and the value of TA and salinity measured on that day. TA and salinity values were assumed to be representative of the conditions of the whole day they were measured on. The calculation was performed using the program CO2SYS v25^34^ and the R package *seacarb* v3.2.16^35^, following Nisumaa et al*.*^36^. Briefly, values of TA, salinity, pH_NBS_, and temperature were used to first calculate total dissolved inorganic carbon (DIC) in CO2SYS, with carbonic acid dissociation constants from Mehrbach et al*.*^37^ refit by Dickson et al*.*^38^, which was subsequently used in *seacarb* to calculate carbonate chemistry parameters, with carbonic acid dissociation constants from Lueker et al*.*^39^. Our approach is suitable for biological OA experiments with treatments that have differences larger than 100 µatm *p*CO_2_^40^ and allowed us to account for the diel oscillation of pH in the experimental tanks.

### Microsensor measurements

Microsensor measurements were conducted in a flume (length 118 cm, width 18 cm, water depth 19 cm) with unidirectional recirculating flow, created by a circulating pump (ES-28, Aqualight, Germany). Flow straighteners were placed up- and downstream of the measurement section (upstream: PVC grid with length of 10.5 cm and 1.3 × 1.3 cm openings, attached to a layer of nylon net with 500 µm pore size; downstream: PVC grid with length of 2.5 cm and 1.3 × 1.3 cm openings). Coral fragments were measured 9.5 cm above the flume bottom, placed on a PVC grid and at an approximate distance of 30 cm from the upstream flow straightener, and were thus assumed to be within the mean bulk flow of the flume. Flow velocity in the flume was measured at the position of the coral fragment for the microsensor measurements using an electromagnetic water flow meter (OTT MF pro, OTT Hydromet GmbH, Germany), and bulk flow velocities of 2 and 6 cm s^−1^ were accomplished by defining different settings for the circulating pump. To estimate the characteristics of the flow entering the flume for the two flow velocities, the dimensionless Reynolds number (Re) was calculated as Re = uW/v from the flow velocity (u), the hydraulic diameter of the flume (W), and the kinematic viscosity (v) of seawater at 26 ºC and salinity 35^41^. To characterise the flow around the coral fragments within the flume under the two flow velocities, Re was calculated (where W is the coral height^5^) based on the average height of coral fragments (3 cm) at the time of microsensor measurements. In the flume, the velocities of 2 and 6 cm s^−1^ corresponded to Re_flume_ of 4006 and 12,019, respectively, indicating that flume bulk flow was transitionally turbulent at the low velocity and fully turbulent at the moderate velocity^42^. Around the coral fragments, however, Re_coral_ was 650 and 1950 for the low and moderate flow velocities, respectively, indicating that the flow experienced by the coral fragments was laminar^42^ for both flows. Light in the flume was provided by two T5 bulbs (80 W, Aqua-Science, Germany) producing a light intensity of 203 ± 9 µmol photons m^−2^ s^−1^ (n = 17), measured before and after the 19-day measurement session of microsensor profiles. Water temperature and pH in the flume were monitored and maintained in the same way as in the experimental tanks (see above). Seawater carbonate chemistry in the flume was calculated using TA values measured during the period of microsensor measurements and daily recording of pH, temperature, and salinity.

O_2_ and pH profiles were measured separately using a Unisense microprofiling system (Unisense, Denmark). Profiles of dissolved O_2_ concentration were measured using Clark-type O_2_ microelectrodes (tip diameter 20–30 µm, spatial resolution 25 µm, 90% response time < 4 s; OX-25, Unisense, Denmark) calibrated daily with air-saturated seawater and anoxic seawater prepared using yeast, following manufacturer’s instructions. pH profiles were measured using a pH microelectrode (tip diameter 40–60 μm, spatial resolution 75 µm, 90% response time < 10 s; pH-50, Unisense, Denmark) and an external reference electrode (Radiometer Analytical), calibrated daily with NBS buffers. Values of pH_NBS_ were converted to pH_T_ using equations from Millero^43^ and Takahashi^44^.

Microelectrodes were connected to a microsensor multimeter (Unisense, Denmark), whose signals were read on a PC using a two-channel A/D converter (ADC-216, Unisense, Denmark). Profiles were performed using a motorised microprofiling system (Unisense, Denmark), by carefully placing the tip of the sensor on the coral surface and moving it up in a series of steps within the gradient (expected range: 200–300 µm) and in bulk seawater, following a protocol developed during prior test runs to ensure that the profile structure was captured for the three species and keep measurement time similar across species. To better resolve the profiles, we used step sizes that were below the spatial resolution at times and thus, integrate values from below steps. Profiles of O_2_ were done in steps of 10–20 µm. At the height of 100 µm above the coral surface, if the electrochemical signal was still close to the value measured at the coral surface, the step size was increased to 30–40 µm until the signal was within 70–80% of the signal corresponding to bulk seawater level, after which the profile was continued with steps of 10–20 µm until the bulk seawater level was reached. In fragments with an estimated larger CBL (thickness > 300 µm), the step size at the height of 100 µm was increased to 40–60 µm until the signal was within 70–80% of the level of bulk seawater. Five coral fragments (four fragments of *A. cytherea* and one of *P. cylindrica*) presented a particularly small O_2_ CBL (thickness < 70 µm) and thus, the first step of their O_2_ profile was done within 5 µm of the coral surface. Profiles of pH were performed in steps of 30–60 µm, except in fragments with a small H^+^ CBL (thickness < 100 µm), for which profiles were done in steps of 20–40 µm. Upon reaching the bulk seawater level, the profiles of O_2_ and pH continued in steps of 50 µm for the first three measurements in bulk seawater, after which profiles continued in steps of 100–200 µm up to 500 µm above the coral surface or up to 1,000 µm above the coral surface for fragments with a larger CBL (thickness > 300 µm). Sensor positioning and data acquisition were performed using the software SensorTrace Profiling (v3.1, Unisense, Denmark, https://unisense.com). At each step, values were recorded for 30–60 s with a waiting period of 3 s after moving the sensor. A total of 356 profiles were recorded. O_2_ profiles were measured in all species under control and OA treatment (n = 72 profiles per species). pH profiles were measured in *A. cytherea* from the control and OA treatment (n = 68), but in *P. verrucosa* and *P. cylindrica* only from the OA treatment (n = 36 per species) due to logistical constraints. Measurements were performed on the top upstream side of coral fragments (Supplementary Fig. S1) after they had been acclimated to flume light and flow conditions (light, 10 min; darkness, 5 min; flow, 10 min), and it was ensured that steady-state conditions (i.e., stable electrochemical signal of O_2_ or pH) had been reached before starting each profile. The chosen acclimation times did not yield visibly different results to longer acclimation (dark, 25 min; flow, 45 min) in prior test runs and fulfilled the need to minimise differences in OA-exposure by allowing the measurement of the large number of profiles within a relatively short period of time. Profiles were measured sequentially under both flow velocities in light and darkness, on the same spot for each coral fragment. The measuring spot was constantly monitored during all profiles using a stereo microscope (Stemi 508, Carl Zeiss AG, Germany) to avoid artefacts due to tissue movement or polyp interaction.

### Calculation of boundary layer traits

O_2_ concentration profiles were used to calculate total thickness of the O_2_ CBL, diffusive flux through the CBL, and O_2_ concentration change at the coral surface relative to bulk seawater (surface ∆O_2_). Thickness of pH gradients across the CBL and pH change at the coral surface relative to bulk seawater (surface ∆pH) were calculated using pH profiles, with pH converted to H^+^ concentration. The CBL thickness was calculated by extrapolating the linear concentration gradient in the CBL to the bulk seawater concentration of the free-flow region^7^, and defined the distance between the coral surface and the outer limit of the CBL. O_2_ flux was calculated using Fick’s first law of diffusion^7,45^ with O_2_ diffusion coefficient of 2.29 × 10^−5^ cm^2^ s^−1^ at 26.0 °C and a salinity of 35^41^. Ciliary vortices at the coral surface may modify the O_2_ transport in the lower part of the CBL, creating S-shaped profiles with shape variability dependent on their position within the vortex^13,46^, which were present in our data. In addition, some profiles in our study presented multiple linear concentration gradients within the CBL, giving them a more complex structure than S-shaped profiles. Within these complex profiles, O_2_ concentration monotonically approached bulk seawater O_2_ values, and this type of profiles is characterised in detail in Martins et al*.*^47^. Complex profiles constituted 7% of all measured profiles and were present in all species (Supplementary Table S3). CBL thickness and O_2_ flux of these profiles were calculated using the upper linear gradient of the profiles (i.e., the linear gradient preceding the reaching of bulk seawater concentration), following Pacherres et al*.*^46^. Additional details on the rationale for this approach are provided in Supplementary Text. Thickness values were pooled over light conditions and profile shapes, and effects associated with complex profiles are visualised in the supplementary material (Supplementary Fig. S2). Surface ∆O_2_ and ∆pH were calculated from the discrete values at the coral surface and bulk seawater. Variation between light and darkness in surface O_2_ concentration and pH was estimated by calculating the difference between light and dark of surface ∆O_2_ and ∆pH in absolute values. The flow ratio for O_2_ CBL traits was calculated as the ratio of values under low flow to moderate flow, pooled over treatments and light conditions.

### Statistical analysis

All statistical analyses were performed in R v.4.1.0^48^ using RStudio v1.4.1106^49^. All plots were produced using the R package *ggplot2*^50^. Changes in the CBL traits of the three studied coral species were investigated using linear mixed-effects models (LMMs). To test differences between species in O_2_ CBL traits we used LMMs with species (3 levels: *A. cytherea*, *P. verrucosa*, and *P. cylindrica*) as a fixed factor. Differences between species in H^+^ CBL traits were assessed considering coral fragments from the OA treatment because pH profiles were measured only under OA for some species (see above). The effects and interaction of flow and OA on O_2_ CBL traits (within each species) and on H^+^ CBL traits of *A. cytherea* were examined using LMMs constructed for each species with flow (2 levels: low and moderate) and treatment (2 levels: control and OA) as fixed factors in a fully crossed design. The effects of flow on H^+^ CBL traits of *P. verrucosa* and *P. cylindrica* were investigated using LMMs constructed for each species with the same structure as above, but without the factor of treatment. All models were constructed with coral fragment identity (ID), coral colony, day of measurement, and tank as random factors, except when the factor had near-zero variance, and treatment was additionally incorporated into global models as a random factor following the same guideline. Models were selected considering AIC, BIC and ﻿R^2^ values. LMMs were performed using the R package *lme4*^51^. Model validation was performed by graphically assessing homogeneity and normality assumptions. To meet model assumptions, we applied a log-transformation to O_2_ CBL thickness (global model with all species included) and a square-root transformation to surface ∆pH values of *P. cylindrica* in darkness. The numerical output of LMMs was extracted using the R package *sjPlot*^52^ and is provided with model formulas in Supplementary Tables S4, S5. We then computed type-II ANOVA tables of the fixed effects of LMMs using Kenward-Roger approximation for the degrees of freedom in the R package *car*^53^. Type II sums of squares was selected to compute ANOVAs, following recommended protocol for assessing main effects individually in the absence of interactions^54,55^. Post hoc analyses were performed using estimated marginal means with Bonferroni adjustment of p-values, using the R package *emmeans*^56^. In addition, the effect size of flow effects was evaluated for O_2_ CBL thickness, H^+^ CBL thickness, surface ∆O_2_, and surface ∆pH using the R package *dabestr*^57^. The relationship between O_2_ CBL flux and surface ∆pH was investigated for each species in the OA treatment, pooled over flow conditions, using Pearson correlations.

Differences in seawater chemistry between experimental pH treatments were tested using daily mean values (n = 72) from days with TA measurements, and the same approach as above (LMM-ANOVA) with treatment as a fixed factor (2 levels: control and OA) and tank and date as random factors.

## Results

### Seawater conditions

During the four months of preconditioning, pH was significantly higher in the control treatment at 7.97 ± 0.13 (mean ± 1 SD; daily range: 7.77–8.19) than in the OA treatment at 7.77 ± 0.14 (range: 7.58–7.99) (LMM-ANOVA, F = 231, p < 0.001) and was similar across replicate tanks (Supplementary Table S6). Both treatments showed a diel oscillation of 0.4 pH units (Table 1). *p*CO_2_ in the control at 497 ± 190 µatm (range: 249–810 µatm) was significantly different from values in the OA treatment at 844 ± 333 µatm (range: 435–1,329 µatm) (LMM-ANOVA, F = 217, p < 0.001), as were other seawater carbonate parameters (Supplementary Table S7). Total alkalinity and temperature were similar between treatments (LMM-ANOVA, F < 0.1/F = 3.6, p > 0.05; Table 1). During the microsensor measurements, pH in the flume was on average 8.02 ± 0.02 and 7.77 ± 0.03 for control and OA treatment, respectively, and seawater parameters resembled the experimental treatments (Supplementary Table S8). In addition, seawater O_2_ was similar during measurements (Supplementary Table S8), with an average of 240 ± 8 µM across treatments and a variation of ± 2 µM within an individual day, indicating that the sequential measurement of profiles did not modify bulk seawater O_2_ concentration.

**Table 1:** Seawater chemistry during four-month preconditioning to a control (ambient pH) and ocean acidification treatment.

Parameter	Control	Ocean Acidification
Salinity	34.6 ± 0.4 (36)	34.6 ± 0.4 (36)
Temperature (ºC)	25.8 ± 0.3 (1,406)	26.0 ± 0.2 (1,224)
pH_T_	7.97 ± 0.13 (1,406)	7.77 ± 0.14 (1,224)
Daily Minimum pH_T_	7.77 ± 0.12 (12)	7.58 ± 0.12 (12)
Daily Maximum pH_T_	8.19 ± 0.04 (12)	7.99 ± 0.06 (12)
TA (µmol kg^-1^)	2,155 ± 53 (43)	2,156 ± 58 (39)
*p*CO_2_ (µatm)	497 ± 190 (1,406)	844 ± 333 (1,224)
Daily Minimum *p*CO_2_ (µatm)	249 ± 32 (12)	435 ± 90 (12)
Daily Maximum *p*CO_2_ (µatm)	810 ± 215 (12)	1,329 ± 370 (12)
*ƒ*CO_2_ (µatm)	496 ± 189 (1,406)	841 ± 332 (1,224)
DIC (µmol kg^-1^)	1,911 ± 80 (1,406)	2,008 ± 79 (1,224)
CO_2_ (µmol kg^-1^)	14 ± 5 (1,406)	23 ± 9 (1,224)
${\text{HCO}}_{3}^{-}$(µmol kg^-1^)	1,717 ± 112 (1,406)	1,858 ± 98 (1,224)
${\text{CO}}_{3}^{2-}$(µmol kg^-1^)	180 ± 44 (1,406)	127 ± 35 (1,224)
Ω_ar_	2.88 ± 0.71 (1,406)	2.02 ± 0.56 (1,224)
Ω_ca_	4.36 ± 1.07 (1,406)	3.06 ± 0.84 (1,224)

Partial pressure of CO_2_ (*p*CO_2_), fugacity of CO_2_ (*ƒ*CO_2_), dissolved inorganic carbon (DIC), CO_2_, ${\text{HCO}}_{3}^{-}$, ${\text{CO}}_{3}^{2-}$, aragonite saturation (Ω_ar_), and calcite saturation (Ω_ca_) were calculated using pH and temperature values of a whole day and the value of total alkalinity (TA) and salinity measured on that day. Values are expressed as mean ± 1 SD with sample size (*n*) of measured parameters (salinity, temperature, pH, TA) and calculated parameters (*p*CO_2_, *ƒ*CO_2_, DIC, CO_2_, ${\text{HCO}}_{3}^{-}$, ${\text{CO}}_{3}^{2-}$, Ω_ar_, Ω_ca_). pH_T_, pH on the total scale.

### Effects of water flow and ocean acidification on boundary layer thickness

The thickness of the CBL in light and darkness showed the same patterns across species and in response to flow velocities and treatments (Table 2). Thickness of the O_2_ CBL differed between species (pooled over light, flow, and treatment; LMM-ANOVA, F = 4.5, p < 0.05), with an overall thinner CBL in *A. cytherea* compared to the rather similar thick CBL in *P. verrucosa* and *P. cylindrica* (Table 2, Supplementary Table S9). The O_2_ CBL was generally thicker under low flow compared to moderate flow but the effect size differed between species (Supplementary Fig. S3A). Thickness increased, respectively, by 61 and 55% in *A. cytherea* and *P. verrucosa* (LMM-ANOVA, F = 53.9/F = 62.2, p < 0.001), whereas in *P. cylindrica* it was 159% thicker (LMM-ANOVA, F = 65.3, p < 0.001) (Supplementary Table S10). Alternatively, *A. cytherea* and *P. verrucosa* presented a low-to-moderate flow ratio of 1.61 and 1.55, respectively, for O_2_ CBL thickness, while the O_2_ CBL of *P. cylindrica* under low flow was 2.58 times as thick as under moderate flow.

**Table 2:** Quantitative traits of the concentration boundary layer (CBL) measured in *Acropora cytherea*, *Pocillopora verrucosa*, and *Porites cylindrica*, in light or darkness combined with low flow (2 cm s^-1^) or moderate flow (6 cm s^-1^), under control or ocean acidification (OA) treatments. Values are expressed as mean ± 1 SD, with measurement replication (n).

Species	Flow	Treatment	Light	O_2_ CBL Thickness (µm)	H^+^ CBL Thickness (µm)	O_2_ CBL Thickness (µm)	H^+^ CBL Thickness (µm)	Surface ∆O_2_ (µM)	Surface ∆pH	O_2_ Flux (µmol cm^-2^ h^-1^)
*Acropora cytherea*	Low	Control	Light	124 ± 75 (72)	94 ± 53 (32)	104 ± 29 (9)	119 ± 38 (8)	73.8 ± 25.2 (9)	0.05 ± 0.02 (8)	0.53 ± 0.16 (9)
	Low	Control	Dark			101 ± 33 (9)	102 ± 80 (8)	–45.7 ± 16.5 (9)	–0.02 ± 0.01 (8)	–0.38 ± 0.13 (9)
	Moderate	Control	Light			79 ± 56 (9)	88 ± 46 (8)	67.2 ± 34.6 (9)	0.03 ± 0.02 (8)	0.67 ± 0.25 (9)
	Moderate	Control	Dark			81 ± 46 (9)	65 ± 28 (8)	–45.4 ± 25.2 (9)	–0.02 ± 0.01 (8)	–0.53 ± 0.23 (9)
	Low	OA	Light		107 ± 54 (36)	202 ± 101 (9)	137 ± 45 (9)	73.4 ± 25.9 (9)	0.05 ± 0.03 (9)	0.32 ± 0.16 (9)
	Low	OA	Dark			203 ± 92 (9)	120 ± 54 (9)	–46.1 ± 14.3 (9)	–0.03 ± 0.01 (9)	–0.23 ± 0.12 (9)
	Moderate	OA	Light			108 ± 45 (9)	70 ± 25 (9)	57.0 ± 29.4 (9)	0.02 ± 0.01 (9)	0.41 ± 0.28 (9)
	Moderate	OA	Dark			111 ± 44 (9)	100 ± 68 (9)	–40.5 ± 17.4 (9)	–0.01 ± 0.00 (9)	–0.35 ± 0.13 (9)
*Pocillopora verrucosa*	Low	Control	Light	162 ± 75 (72)	n/a	201 ± 93 (9)	n/a	94.7 ± 42.2 (9)	n/a	0.68 ± 0.44 (9)
	Low	Control	Dark			209 ± 75 (9)	n/a	–87.5 ± 18.5 (9)	n/a	–0.45 ± 0.20 (9)
	Moderate	Control	Light			118 ± 60 (9)	n/a	58.7 ± 43.4 (9)	n/a	0.48 ± 0.20 (9)
	Moderate	Control	Dark			108 ± 37 (9)	n/a	–72.5 ± 23.8 (9)	n/a	–0.63 ± 0.21 (9)
	Low	OA	Light		106 ± 72 (36)	211 ± 82 (9)	148 ± 99 (9)	101.9 ± 40.6 (9)	0.02 ± 0.04 (9)	0.51 ± 0.29 (9)
	Low	OA	Dark			166 ± 29 (9)	144 ± 55 (9)	–86.2 ± 25.1 (9)	–0.09 ± 0.05 (9)	–0.45 ± 0.13 (9)
	Moderate	OA	Light			155 ± 64 (9)	57 ± 37 (9)	52.6 ± 22.3 (9)	0.01 ± 0.01 (9)	0.37 ± 0.21 (9)
	Moderate	OA	Dark			129 ± 74 (9)	76 ± 31 (9)	–67.1 ± 16.4 (9)	–0.05 ± 0.04 (9)	–0.51 ± 0.15 (9)
*Porites cylindrica*	Low	Control	Light	192 ± 147 (72)	n/a	282 ± 196 (9)	n/a	96.6 ± 28.5 (9)	n/a	0.39 ± 0.23 (9)
	Low	Control	Dark			287 ± 170 (9)	n/a	–57.2 ± 15.0 (9)	n/a	–0.20 ± 0.16 (9)
	Moderate	Control	Light			102 ± 34 (9)	n/a	78.8 ± 29.8 (9)	n/a	0.65 ± 0.28 (9)
	Moderate	Control	Dark			106 ± 39 (9)	n/a	–44.3 ± 15.8 (9)	n/a	–0.37 ± 0.15 (9)
	Low	OA	Light		103 ± 51 (36)	252 ± 140 (9)	119 ± 44 (9)	100.1 ± 53.5 (9)	0.04 ± 0.02 (9)	0.43 ± 0.45 (9)
	Low	OA	Dark			287 ± 166 (9)	128 ± 62 (9)	–44.0 ± 12.7 (9)	–0.05 ± 0.03 (9)	–0.17 ± 0.13 (9)
	Moderate	OA	Light			117 ± 81 (9)	81 ± 50 (9)	83.1 ± 37.3 (9)	0.02 ± 0.01 (9)	0.78 ± 0.60 (9)
	Moderate	OA	Dark			104 ± 58 (9)	86 ± 36 (9)	–37.9 ± 11.3 (9)	–0.02 ± 0.01 (9)	–0.34 ± 0.08 (9)

**Figure 1:**
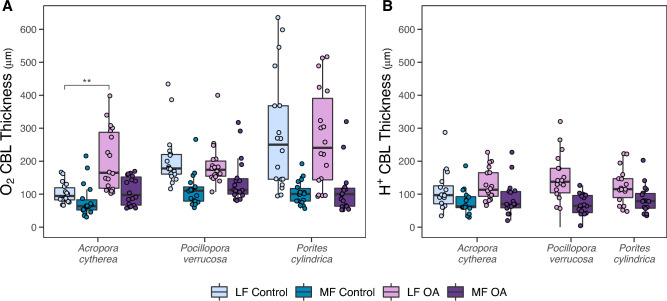
Effects of water flow and ocean acidification (OA) on the thickness of the concentration boundary layer (CBL) of *Acropora cytherea*, *Pocillopora verrucosa*, and *Porites cylindrica*. (**A**) O_2_ and (**B**) H^+^ CBL thickness after four months in a control and OA treatment and measured in light or darkness combined with low flow (LF, 2 cm s^-1^) and moderate flow (MF, 6 cm s^-1^). Values are presented pooled over light and dark conditions. Boxes represent the first and third quartiles with lines as medians and whiskers as the minimum and maximum values or up to the 1.5 * interquartile range (IQR), whichever is reached first. Stars indicate significant differences between the control and OA treatment within each flow condition (p < 0.01**, from linear mixed-effects models with ANOVA).

The effect of OA differed between species (Fig. 1A). In *A. cytherea*, the O_2_ CBL thickness increased under OA (LMM-ANOVA, flow–OA interaction, F = 20.3, p < 0.001) and doubled in the OA treatment compared to the control under low flow, but under moderate flow it remained stable between treatments (Table 2, Supplementary Table S9). In *P. verrucosa* and *P. cylindrica*, O_2_ CBL thickness was similar between treatments (LMM-ANOVA, F < 0.1, p > 0.05; Supplementary Table S11). CBL thickness was generally higher in complex profiles (Supplementary Fig. S2), but OA effects were not dependent on the presence of this profile type (Supplementary Table S12).

The H^+^ CBL was generally thinner than the O_2_ CBL and we did not see differences between the three species (comparing OA treatment pooled over flow and light; LMM-ANOVA, F < 0.1, p > 0.05; Table 2). Again, the H^+^ CBL was thicker under low flow than moderate flow (Fig. 1B) in all species (LMM-ANOVA, *A. cytherea*: F = 14.2, p < 0.001, *P. verrucosa*: F = 36.0, p < 0.001, *P. cylindrica*: F = 21.2, p < 0.001; Supplementary Table S10). In contrast to OA effects on the O_2_ CBL thickness, the H^+^ CBL of *A. cytherea* had similar thickness in the control and OA treatments (pooled over flow and light; LMM-ANOVA, F = 0.2, p > 0.05; Fig. 1B, Table 2).

### O_2_ and pH changes at the coral surface

**Figure 2:**
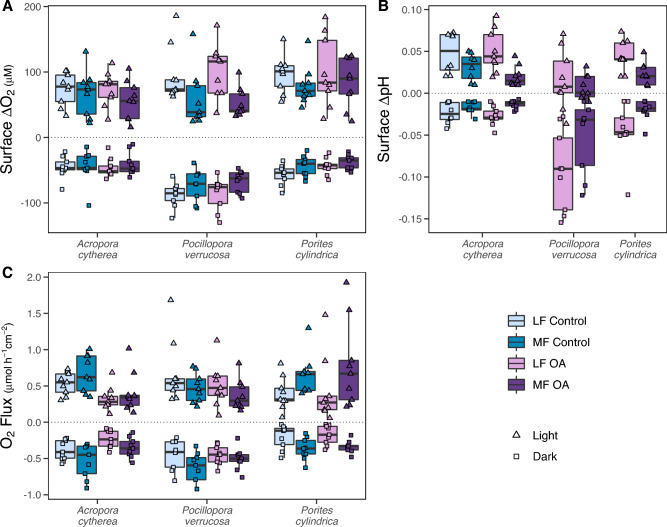
Effects of water flow and ocean acidification (OA) on traits of the concentration boundary layer (CBL) of *Acropora cytherea*, *Pocillopora verrucosa*, and *Porites cylindrica*. (**A**) O_2_ concentration change at the coral surface relative to bulk seawater (surface ∆O_2_), (**B**) pH change at the coral surface relative to bulk seawater (surface ∆pH), and (**C**) O_2_ flux, after four months in a control and OA treatment and measured in light or darkness combined with low flow (LF, 2 cm s^-1^) and moderate flow (MF, 6 cm s^-1^). Boxes represent the first and third quartiles with lines as medians and whiskers as the minimum and maximum values or up to the 1.5 * interquartile range (IQR), whichever is reached first. No significant interactive effects of flow and OA were observed.

O_2_ concentration at the coral surface in light was super-saturated with respect to bulk seawater with similar magnitude across species (LMM-ANOVA, F = 0.9, p > 0.05), which was on average 78.2 ± 37.6 µM above seawater concentration (pooled over flow and treatment). In darkness, however, surface ∆O_2_ differed between species (LMM-ANOVA, F = 10.6, p < 0.05), with larger under-saturation in *P. verrucosa* than the similar reduction in surface O_2_ in *A. cytherea* and *P. cylindrica* (Supplementary Tables S9, S13). *Pocillopora verrucosa* thus presented a larger variation in surface O_2_ concentration between light and darkness (pooled over flow and treatment, 155.31 ± 52.83 µM) than *A. cytherea* (112.26 ± 44.06 µM) and *P. cylindrica* (135.51 ± 45.17 µM). Response to flow and OA in surface ∆O_2_ showed similar patterns in light and dark conditions (Fig. 2A, Table 2). Although average differences between flows were relatively small (Supplementary Table S10), ∆O_2_ was larger under low flow (light/dark, LMM-ANOVA, *A. cytherea*: F = 9.9, p < 0.01; *P. verrucosa*: F = 38.6/F = 17.8, p < 0.001/0.01; *P. cylindrica*: F = 11.2/F = 4.9, p < 0.01/0.05), except in *A. cytherea* in dark (LMM-ANOVA, F = 1.8, p > 0.05), with notable uncertainty in effect sizes for *A. cytherea* and *P. verrucosa* in light (Supplementary Fig. S3C). Overall, these differences resulted in a higher ratio of low to moderate flow in *P. verrucosa* (1.48) than *A. cytherea* (1.14) and *P. cylindrica* (1.22).

In contrast, OA did not significantly modulate surface ∆O_2_ across species (light/dark, LMM-ANOVA, *A. cytherea*: F = 0.9/F = 0.3, p > 0.05; *P. verrucosa*: F < 0.1/F = 0.1, p > 0.05; *P. cylindrica*: F < 0.1/F = 2.7, p > 0.05; Supplementary Table S11).

Generally, pH was elevated at the coral surface in light conditions and reduced in darkness compared to bulk seawater (Fig. 2B). However, the magnitude differed between species in light and dark conditions (comparing OA treatment pooled over flow; LMM-ANOVA, F = 4.5/F = 88.5, p < 0.05/0.001). *Pocillopora verrucosa* showed the lowest pH elevation in light but the strongest decrease in darkness compared to similar pH elevation in light and decrease in darkness in *A. cytherea* and *P. cylindrica* (Supplementary Table S11). Flow significantly affected the pH change in both *A. cytherea* and *P. cylindrica* with larger pH elevation in the light (LMM-ANOVA, F = 35.6/F = 24.4, p < 0.001/0.01) and larger decrease in darkness (LMM-ANOVA, F = 11.9/F = 9.9, p < 0.01/0.05) under low flow compared to moderate flow (Supplementary Table S10). In *P. verrucosa* the effect was less clear, with similar values between flows under light conditions (LMM-ANOVA, F = 1.1, p > 0.05) and a weak pH reduction in darkness under low flow compared to moderate flow (LMM-ANOVA, F = 9.9, p < 0.05; Supplementary Fig. S3D) (Supplementary Table S10). Overall, surface pH of *P. verrucosa* under OA varied between light and darkness by 0.11 ± 0.05 and 0.06 ± 0.05 pH units in low and moderate flow, respectively, which was similar to the light–dark variation observed in *A. cytherea* (low flow: 0.08 ± 0.04; moderate flow: 0.03 ± 0.01 pH units) and *P. cylindrica* (low flow: 0.09 ± 0.05; moderate flow: 0.04 ± 0.02 pH units). As observed for surface ∆O_2_, the relative change in surface pH of *A. cytherea* was also similar between the control and OA treatments (light/dark, LMM-ANOVA, F = 0.3/F < 0.1, p > 0.05; Supplementary Table S11) and presented a light–dark variation of 0.06 ± 0.03 pH units in both treatments (pooled over flow velocities).

### O_2_ flux across the coral boundary layer

O_2_ flux rates across the CBL were overall similar among species in light (LMM-ANOVA, F < 0.1, p > 0.05) but not in dark (LMM-ANOVA, F = 13.9, p < 0.001) (pooled over flow and treatment), where mean flux values were higher in *P. verrucosa* compared to the similar flux in *A. cytherea* and *P. cylindrica* (Supplementary Tables S9, S13). Like CBL thickness and surface ∆O_2_, responses to flow and OA in O_2_ flux generally showed similar patterns under light and dark conditions. O_2_ flux was generally lower under low flow compared to moderate flow (light/dark, LMM-ANOVA, *A. cytherea*: F = 6.6/F = 14.6, p < 0.05/0.01; *P. verrucosa*: F = 16.7, p < 0.01; *P. cylindrica*: F = 9.3/F = 13.1, p < 0.01), except in *P. verrucosa* in light (LMM-ANOVA, F = 3.4, p > 0.05) (Fig. 2C). Differences between flows were largest in *P. cylindrica* (Supplementary Table S10), which presented the lowest low-to-moderate flow ratio (0.56) compared to *A. cytherea* (0.75) and *P. verrucosa* (1.06), showcasing thus the same pattern as O_2_ CBL thickness but with the opposite direction. The response to OA in O_2_ flux also displayed the same species-specific patterns as O_2_ CBL thickness. While in *A. cytherea* O_2_ flux was reduced in the OA treatment compared to the control (light/dark, LMM-ANOVA, F = 7.3/F = 6.1, p < 0.05), it remained similar between treatments in *P. verrucosa* and *P. cylindrica* (light/dark, LMM-ANOVA, *P. verrucosa*: F = 1.7/F = 0.6, p > 0.05; *P. cylindrica*: F = 0.1/F = 0.5, p > 0.05) (Supplementary Table S11). Similarly, to the assessments in O_2_ CBL thickness, the flux of complex profiles fell within the lower range of the observed fluxes but did not drive OA responses (Supplementary Fig. S4).

Coral O_2_ flux in light was not correlated with change in surface pH in any species, while in darkness it was negatively correlated in *A. cytherea*, but not in *P. verrucosa* or *P. cylindrica* (Supplementary Fig. S5).

## Discussion

**Figure 3﻿:**
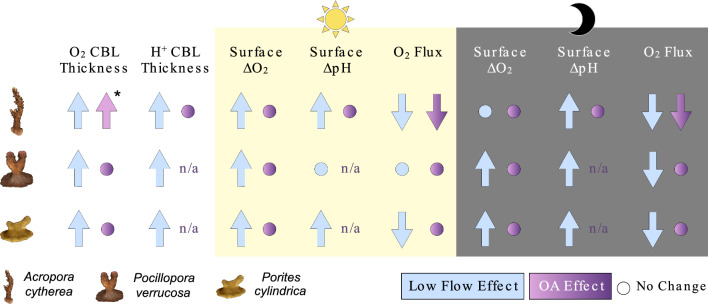
Summary diagram of the effects of water flow and ocean acidification (OA) on traits of the concentration boundary layer (CBL) of *Acropora cytherea*, *Pocillopora verrucosa*, and *Porites cylindrica*. Effects on CBL thickness are presented pooled over light conditions, while change in surface O_2_ concentration (surface ∆O_2_) and pH (surface ∆pH) relative to bulk seawater, and O_2_ flux are presented in light and darkness. Asterisk indicates OA effects present exclusively under low flow. n/a, not available.

Our study shows that the coral CBL is strongly influenced by changes in water flow, while OA may have weak or null effects (Fig. 3), even after prolonged exposure. In this study, CBL traits were distinct between species but low flow thickened boundary layers across species and enhanced the relative change of surface O_2_ and pH, compared to moderate flow. In contrast, CBL traits remained largely stable under OA, except the absolute values of surface pH, which were considerably lower under OA. Finally, O_2_ flux across the CBL was strongly influenced by changes in its thickness.

CBL traits differed between the three species investigated in this study. Thickness of the O_2_ CBL, in particular, was the most species-differentiating trait and was thinnest in *A. cytherea*. Although variation in CBL traits has been documented previously, branching species are underrepresented in microsensor studies and comparative studies have generally focused on the differences between large- and small-polyped species^12^. Our dataset builds upon this knowledge and shows that CBL variation exists even among small-polyped species. In addition, a previous report of similar O_2_ CBL thickness between *Stylophora pistillata* and *Porites lobata*^10^ supports our finding of similar CBL thickness between pocilloporids and poritids.

While our data is in line with the notion that morphologically complex corals have thinner CBLs^58^ due to the potential of complex geometries to isolate ciliary flows from bulk water flow^13^, other factors could also underlie this variation. CBL thickness may decrease when cilia activity is arrested^13^, suggesting that the thinner CBL of *A. cytherea* in this study could also be due to a lower abundance and/or beating frequency of cilia. Acroporids are reported to have weak feeding and cleaning ciliary currents^59,60^, which could indicate that ciliary surface ventilation is also weaker in *Acropora* spp. than other taxa. Differences in cilia traits and activity among reef-building corals, however, are poorly characterised to date.

The effects of water flow on CBL traits in this study were consistent across species and molecules. Boundary layers of both O_2_ and pH gradients were thicker under low flow compared with moderate flow in all tested species, which is in agreement with the known effects of reduced flow on the CBL (e.g.^9,12^). However, the magnitude of this increase differed between species in our study, which indicates that the coral response to flow in CBL thickness is influenced by additional species-specific factors. Here, the largest flow ratio was observed in *P. cylindrica*. Polyp size is known to be similar across the three species tested^61^, and *P. verrucosa* and *P. cylindrica* have similar polyp densities^62,63^, with no differences in cilia activity reported to date between these two species. Therefore, the different flow response of *P. cylindrica* could be associated with other small-scale differences in the topography of the coral tissue surface and with different polyp behaviour. For instance, *Porites* spp. have been described to have greater powers of polyp expansion than most small-polyped species^64,65^ and water flow can affect polyp expansion^66,67^. During our measurements, polyp expansion appeared to be more prevalent and to occur to a greater extent in *P. cylindrica* under low flow than in the other species (quantitative characterisation not available). Furthermore, mathematical models predict thicker CBLs and lower flux for expanded polyps^68^, which is in line with the patterns observed in this study. Nonetheless, future studies using computational fluid dynamics may be able to further disentangle the relationship between flow velocity and CBL thickness across small-polyped coral species.

In contrast to flow effects, we found that CBL thickness remained stable under OA, except in *A. cytherea*, which experienced a thickening of the O_2_ CBL under OA with low flow. Changes in coral microtopography and surface cilia activity could potentially underlie the thickening of the O_2_ CBL of *A. cytherea* under OA^12,13^. OA has been shown to alter the internal structure of coral skeletons^20,69^, reduce skeletal density^70^ and septal rugosity^71^, and alter corallite size^72^. However, it remains untested whether long-term OA can alter the roughness of the coral tissue surface enough to affect CBL thickness. OA has also been shown to reduce cilia beat frequency^18^ and gene expression involved in cilia formation in bivalves^73^ as well as decrease formation and stability of flagella in phytoplankton^19^. Thus, changes in surface cilia activity of *A. cytherea* may have occurred under OA, thereby resulting in the thickening of its O_2_ boundary layer. Given that cilia effects on the coral boundary layer are more visible under low flow than under moderate flow^46^, this hypothesis would also explain the presence of an OA effect exclusively under low flow. The presence of the five complex O_2_ concentration profiles in *A. cytherea* under OA with low flow could further point to cilia changes. However, although *P. cylindrica* also presented complex profiles under low flow, these were equally frequent in both treatments. Therefore, the significance of the occurrence of these profiles here remains unclear.

The only other investigation of OA effects on coral CBL thickness to date has been on *Favites* sp. and also found interactive flow and OA effects but with the opposite pattern (i.e., thicker O_2_ CBL under OA with moderate flow than low flow)^16^. Responses to OA at the scale of the CBL may thus be species-specific, with susceptibility potentially associated with differences in surface cilia, and differentially modulated by water flow.

While our results indicate that low flow could modulate the CBL to better isolate the coral colony from acidified bulk seawater, low flow conditions could become less frequent in future reefs. Most surface ocean currents are projected to accelerate with climate change^74^ and sea level rise is predicted to overall increase wave energy in coral reefs^75^. While this effect is likely exacerbated in degraded reefs^76^, the effects of sea level rise also differ among reef types^3^ and vary spatially within single reefs^77^. For instance, reefs with an estimated general increase in wave energy will also present zones where flow velocities decrease^26^. In addition, several major surface ocean currents are also predicted to weaken, including tropical currents that affect coral reefs such as the Indonesian Throughflow^78,79^. Altogether, future changes to reef hydrodynamics make the inclusion of low-flow refuge areas all the more important in coral reef conservation strategies that address OA challenges on coral species.

Although the H^+^ CBL partially isolates the coral from bulk seawater pH and could determine coral susceptibility to OA^80^, it has never been characterised in scleractinian corals under OA. Thus, our study constitutes the first report of the thickness of the coral H^+^ CBL under OA. We found that the H^+^ CBL was similarly thin across species. Given that the three species investigated here are known to differ in their susceptibility to OA, with acroporids generally regarded as more OA-susceptible than pocilloporids^81^ and poritids^82^, our findings suggest that susceptibility may not be associated with the thickness of the H^+^ CBL.

In addition, we found that the thickness of the H^+^ CBL of *A. cytherea* was not modulated by the OA treatment, suggesting that the markedly thin H^+^ CBL could be intrinsic to the tested species. For instance, *Acropora yongei* has been shown to have no H^+^ CBL under ambient pH^17^ (possibly too thin to measure with microsensors). However, other *Acropora* spp. featured a H^+^ CBL thicker than 300 µm^9,58^. The high variability reported for pH boundary layers could be due to spatial heterogeneity of the coral surface, but also due to experimental differences.

In this study, the H^+^ CBL was generally thinner than O_2_ gradients under OA, which contrasts with previous reports of O_2_ and H^+^ CBL with similar thickness under ambient reef conditions^9,58^. The observed dissociation of O_2_ and pH gradients in our study could be due to spatial heterogeneity of the coral surface since O_2_ and pH profiles were measured on separate occasions and thus on different locations of the coral fragment; though, care was taken to perform all measurements in a similar area of the fragment (top area of the upstream side). O_2_ concentration, for instance, has been shown to vary greatly along the coral surface^83,84^. This hypothesis would also explain the absence of a correlation between surface ∆pH and O_2_ flux in this study, despite surface pH being closely linked to photosynthesis and respiration via coral CO_2_ uptake and release^9,85^. However, such a result could also indicate that the CO_2_ used during photosynthesis in the tested corals was not coming from seawater but had metabolic origins^86^. Future studies that simultaneously characterise both O_2_ and pH gradients across the CBL will be crucial to unravel the relationship between these two CBLs under OA, the potential uncoupling of the associated physiological processes (e.g., photosynthesis, respiration, and calcification), and better understand carbon fluxes in scleractinian corals.

The surface microenvironment of reef-building corals is shaped by the dynamic build-up and depletion of metabolic molecules at the coral surface and thus fluctuates drastically between day and night, with O_2_ and pH levels above seawater values during the day and below during the night^9,85^. This behaviour was successfully replicated in our study in light and dark conditions but changes in surface pH relative to bulk seawater were overall small in the tested corals (< 0.1 ∆pH). Although the literature is still limited, such low elevation of surface pH may be typical of some coral taxa under ambient pH, particularly of branching species^16,58^. For instance, under ambient pH and flow velocities similar to our study, *Acropora aspera* has been shown to have a pH elevation of similar magnitude to that recorded here for *A. cytherea*^58^, while no elevation was found in *A. yongei*^17^. As observed here and in previous studies^9,16^, the coral surface microenvironment is further modulated by water flow, with reduced flow velocities inducing larger elevation and depletion of O_2_ and pH levels. Like CBL thickness, the response to flow in surface values also differed in magnitude among species as suggested by the flow ratios observed in this study. However, in surface dynamics, the highest flow ratio was observed in *P. verrucosa*, which could indicate a more responsive physiology to flow changes in this species.

The pH microenvironment at the coral surface is considered a defining factor of coral susceptibility to OA^21^ because elevating surface pH may provide a crucial buffer during daylight that shelters the coral from the acidified bulk seawater^15,87^. In line with this, environmental pH variability can influence coral responses to OA^88,89^, though higher pH during daytime may not be the sole determinant of reduced OA effects on whole-colony physiology^90^. In this study, while the amplitude of light–dark variation of surface pH was similar among species, pH behaviour was not symmetric in light and darkness and differed between species. The smallest pH elevation was observed in *P. verrucosa* under OA, which also had the largest pH reduction in darkness. However, this did not underlie a larger reduction in growth under OA (investigated in a study that assessed the OA-induced metabolic changes in the same coral fragments as studied here^91^), which was overall similar between *P. verrucosa* and *P. cylindrica*, and larger in *A. cytherea*. These results suggest that coral OA susceptibility is not determined by the magnitude of pH fluctuation at the coral surface, consistent with previous findings that greater seawater pH variability does not systematically enhance OA resistance^92^. Still, pH variability effects may depend on physiological response curves^90^ and differ among species^93,94^. Low pH at the coral surface likely alters H^+^ gradients across tissue layers^95,96^. This leads to decreased pH within coral cells^89,97,98^, in the mesoglea above the calcifying cell layer^99^, and of the calcifying fluid^100^, which has been observed in *P. verrucosa* even when calcification remains stable under OA^101^. Therefore, our findings suggest that *P. verrucosa* may undergo large internal pH changes under OA with limited effects on growth, indicating an enhanced capacity for internal pH regulation. However, countering this strong pH reduction in coral tissues may be energetically costly, as shown by trends of decreased lipid content in *P. verrucosa*^102^. Moreover, pH regulation could be additionally aided by increasing tissue thickness under OA, which would further separate the calcifying cell layer and the coral surface, and expand the space where pH is actively regulated. Although not observed in *P. verrucosa*, other pocilloporids have shown tissue thickening under OA^102,103^.

Similarly to *P. verrucosa*, the lack of substantial elevation of surface pH observed here in *P. cylindrica* is remarkable, considering its mild metabolic response to OA^91^. However, given that *P. cylindrica* may maintain a constant pH of the calcifying fluid under OA^104^, acid–base regulation could potentially differ between these two species^97^. Knowledge of these mechanisms and of the pH gradients between tissue layers and extracellular compartments, however, remains limited in scleractinian corals^99^. Furthermore, although *P. cylindrica* does not increase tissue thickness under OA^102^, it has characteristically thicker tissues than *P. verrucosa*^105^, which could underlie a greater ability to counter internal pH changes.

Bulk seawater pH did not affect the relative change in surface pH of *A. cytherea*, meaning that the absolute values of surface pH were considerably lower in the OA treatment than the control. These findings contrast with the higher surface pH elevation under OA (compared to ambient pH) observed previously in large-polyped massive species (*Favites* sp. and *Galaxea fascicularis*), with ∆ values up to 0.8 pH units^15,16^. These differences could be due to morphological variation, which has been previously proposed as an underlying factor of differential pH elevation among coral species^58^. However, although our study shows that under OA surface ∆pH was overall similar between branching species (*A. cytherea* and *P. verrucosa*) and encrusting species with long uprights (*P. cylindrica*)^106^, we are unable to address here the link between morphological complexity and responses to OA due to the lack of H^+^ CBL data for *P. verrucosa* and *P. cylindrica* under ambient pH. Still, given the small relative change in pH in *P. cylindrica* under OA, an OA-enhanced elevation of surface pH seems unlikely. Future studies able to accurately characterise a range of coral morphologies and CBL responses to OA will be key to elucidate the role of morphological complexity and establish whether the response of *A. cytherea* in H^+^ CBL traits is common to small-polyped species.

Overall, our findings demonstrate the absence of a buffer from OA conditions at the coral surface and highlight the importance of pH regulation mechanisms under OA, which, however, may become impaired under heat stress^107^. The pH microenvironment of *P. verrucosa* and *P. cylindrica* recorded here could be specific to OA conditions. Therefore, future studies that characterise the pH microenvironment of these species under both ambient pH and OA will help establish the response of their surface pH to OA. Nonetheless, *Pocillopora damicornis* has also shown surface ∆pH below 0.1 pH units under ambient pH^16^, further supporting our finding that the resilience of pocilloporids to OA does not rely on being sheltered by their boundary layer.

Characterising diffusive fluxes across the CBL provides a comprehensive understanding of coral responses at the CBL scale. The values of O_2_ flux in this study are based on diffusive transport, derived most commonly through the entire CBL and only in a few cases from the upper linear gradient. The fluxes recorded here are in line with previous reports (< 1 µmol cm^−2^ h^−1^; e.g.^9,46^). The assessment of fluxes in corals can become more complicated when cilia activity modulates the flow within the CBL, creating vortices that result in O_2_ concentration profiles with a complex shape^13,46^. Although in our study we did not aim to resolve the microscale flow patterns and conditions within the CBL, the frequency of complex profiles observed here was low (7% of all profiles). This low occurrence is likely due to the moderately high bulk flow velocities used in our study, which are expected to compress surface vortices^13^. Further detailed evaluation of our profiles indicated that there were only small deviations in the fluxes derived from the upper linear gradient of complex profiles. This supports the assumption that these derived fluxes are representative of the total flux across the CBL^46^, but further assessments, including microscale visualisation of the flow patterns within the CBL, are desirable. Such in-depth studies that capture all the mechanisms that modulate CBL dynamics, including molecular diffusion, turbulence, and other unknown mechanisms^13,46,84,108^, will help to build a more robust mechanistic understanding of mass transfer within the coral CBL.

O_2_ flux decreases with decreasing water flow due to the thickening of the CBL^9^. In this study, the O_2_ flux response to short-term exposure to low flow (< 1 h) reflected this same pattern, in both light and darkness. This result is in line with the decreasing effect of reduced flow on whole-colony net photosynthesis and respiration^109,110^. Thus, our findings support the notion that flow may modulate coral photosynthesis and respiration through changes on the boundary layer that affect the mass transfer of O_2_ between the coral and seawater^111^.

OA effects on coral O_2_ flux have been previously investigated in only one other study, which found no changes in light O_2_ flux of *Favites* sp. after 30-min exposure to OA^16^. In our study, we demonstrate that some species may maintain stable O_2_ flux even after prolonged exposure to OA (as observed in *P. verrucosa* and *P. cylindrica*), while others experience a reduction (as seen in *A. cytherea*). In addition, *A. cytherea* was also the only species with OA effects on the thickness of the O_2_ CBL, highlighting the strong relationship between O_2_ flux and CBL thickness. Small-scale changes could, therefore, be one of the underlying factors of the OA-induced decrease in net photosynthesis of the studied coral fragments of *A. cytherea*^91^ and of other acroporids (e.g.^112,113^). If so, photosynthesis of *Acropora* spp. may be mass transfer limited by the thicker CBL under OA, specifically the lower efflux of O_2_. Accumulating O_2_ within the coral during light may increase photorespiration, thereby decreasing photosynthetic activity^110^. In addition, reduced O_2_ flux may have physiological effects other than reduced photosynthesis, such as increased oxidative stress^114^. Overall, our results indicate that in some species reduced photosynthesis under OA could be linked to CBL changes, with potential downstream effects in overall growth due to the reduction in energy supply from the microalgae to the coral host.

Although individual CBL traits could potentially influence whole-colony responses to OA in different ways, the overall limited effects of OA on the CBL in our study, except in surface absolute values, suggest that OA-induced changes in surface values may have the largest influence on whole-colony physiology by altering coral internal gradients. However, since responses at the CBL scale may be species-specific, the pathways and mechanisms underlying whole-colony responses to OA could differ among species.

## Conclusions

Our study reinforces that the coral boundary layer is strongly modulated by changes in water flow and suggests an overall weak to null modulation by OA, even after prolonged exposure. We show that I) while CBL thickness increases under reduced flow, it may remain largely stable under OA. Nonetheless, the coral H^+^ CBL was markedly thin under OA, providing limited buffering against OA conditions. We also demonstrate that II) the relative change of O_2_ and pH at the coral surface may be enhanced by reduced flow but not OA, which, in turn, lowered the absolute value of surface pH. These OA effects contrast with previous findings of OA-induced elevation of surface pH in large-polyped reef-building corals and could thus be specific to small-polyped species. Therefore, our results reveal for the first time that reef-building coral species may lack a significantly differentiated pH microenvironment separating the coral from bulk seawater under OA, indicating that the physiological resilience to OA of some coral taxa may not rely on being sheltered by their boundary layer. Still, other environmental factors, such as reduced water flow, may modulate the CBL to offer some shelter. Finally, we confirm that III) responses in O_2_ flux to flow and OA may reflect the same species-specific patterns as responses in CBL thickness and show that some coral species may maintain O_2_ flux stable under long-term OA.

In this study, however, the assessment of the OA response in H^+^ CBL traits was possible for *A. cytherea* only. Future investigation in more species will help establish whether the H^+^ CBL response observed in *A. cytherea* is common among small-polyped corals. Furthermore, we observed no clear relationship between O_2_ and pH gradients, which may have been due to spatial heterogeneity across the coral surface. Future studies able to simultaneously characterise both O_2_ and pH gradients across the CBL under OA will be crucial to unravel the relationship between these two gradients and the potential uncoupling of the associated physiological processes. Finally, although our data suggest that cilia influence on the CBL was limited in our study, we did not characterise the microscale flow conditions during the measurement of profiles and thus, are unable to resolve their microscale flow context. Future in-depth microsensor studies capable of integrating across the various mechanisms that may modulate CBL dynamics will improve our mechanistic understanding of the processes that govern mass transfer within the coral CBL, particularly the enhancement by vortical ciliary flow. Understanding how small-scale elements and whole-colony physiological responses integrate will be key to explain the diversity of coral responses to OA.

### Supplementary Information

Supplementary Information.

## Data Availability

The datasets generated and analysed in the current study are available in the Figshare repository, https://doi.org/10.6084/m9.figshare.24534343.
