# Supplementary material for: Effects of water flow and ocean acidification on oxygen and pH gradients in coral boundary layer
Source: Sci Rep. 2024 Jun 4;14:12757. doi: 10.1038/s41598-024-63210-9 (PMC11148076; doi:10.1038/s41598-024-63210-9)
Supplement: Supplementary file 1 — Supplementary Information. [file 41598_2024_63210_MOESM1_ESM.pdf]

Supplementary material

Effects of water flow and ocean acidification on oxygen and pH gradients in coral boundary layer

Catarina P.P. Martins*[1], Maren Ziegler[1], Patrick Schubert[1], Thomas Wilke[1], Marlene Wall[2]

[1]Department of Animal Ecology & Systematics, Justus Liebig University Giessen, 35392 Giessen, Germany

[2]GEOMAR Helmholtz Centre for Ocean Research Kiel, 24148 Kiel, Germany

*corresponding author: catarina.padilha-pires-martins@bio.uni-giessen.de

Supplementary Text

In our study, we followed the approach of Pacherres et al.[1] and calculated O_2 flux using the upper linear gradient of the profile. Briefly, we chose this approach because all the calculations of flux of Pacherres et al.[1] are performed using Fick's first law of diffusion (i.e., based on molecular diffusion) and the diffusion coefficient based on temperature and salinity. Additionally, we followed this procedure because in their study Pacherres et al.[1] provided a first proof that the flux in the upper linear gradient of complex profiles (upper flux) is representative of the actual flux across the concentration boundary layer (CBL) of the coral. Specifically, they compared fluxes under the arrested cilia state (calculated using almost the entire profile) and under the active cilia state (calculated using the upper gradient of the profile), which revealed the same flux under both cilia states. This indicates that the flux is independent of the overall underlying transport mechanisms (diffusion vs a combination of diffusion and enhanced transport by vortical movement) across the CBL. In our study, however, we did not characterise the microscale flow conditions surrounding the measuring spot at the coral surface during profile measurement or perform additional measurements under arrested cilia activity. Thus, we are unable to ascertain how much the approach used to derive the O_2 flux of complex profiles deviates from the actual flux. However, in our data, complex profiles were few (Supplementary Table S3) and the CBL was generally thin (see the Results section), which suggests that the effect of ciliary vortices is negligible in our data. Thus, by following Pacherres et al.[1] and using only the linear section and diffusive parts to calculate fluxes we grasp the major transport process that govern the flux in our profiles.

Alternative approaches for the treatment of these complex profiles would be to exclude them from the study or to calculate O_2 flux without considering their profile structure and using only the total CBL thickness and maximum O_2 concentration at the coral surface (i.e., treating them as if all profiles had the same structure). However, we believe that it is important to include these profiles in our study to fully capture the variability of the CBL of the three tested species and their biological responses. Additionally, we categorised all profiles into three main shapes (diffusive, S-shaped, and complex), as outlined in Martins et al.[2], and did not detect extreme deviation associated with the complex profiles (Figure i). Furthermore, while the alternative calculation approach might be a valid option for studies with thick CBLs and strong cilia influence, this was not the case for our study. The limited influence of cilia activity in our data is mostly likely due to the moderately high bulk flow velocities used here, which are expected to compress surface vortices and strongly reduce the effects of cilia movement on the CBL[3]. In line with this, the O_2 flux of the complex profiles derived following both approaches—based on the upper liner gradient of profiles vs based on total CBL thickness and maximum surface O_2 concentration—on average differ by only 0.04 µmol h^{-1} cm^{-2} (n = 23) (Table i). Altogether, this suggests that calculating the O_2 flux of the complex profiles in our study following the approach outlined by Pacherres et al.[1] provides a representative estimation of coral activity and flux.

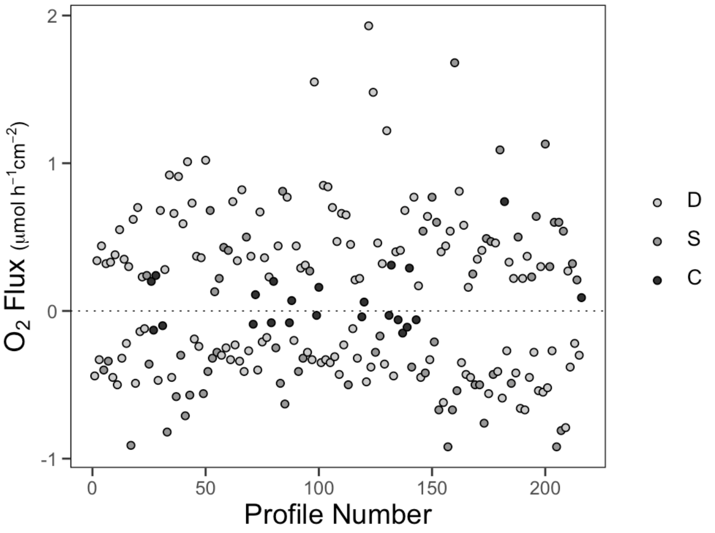

Figure i. Derived O_2 flux values for all measured O_2 concentration profiles, categorised into three main profile shapes (D, diffusive; S, S-shaped; C, complex).

Table i. Slope and respective fluxes of complex profiles calculated based on (1) the upper linear gradient of profiles, and (2) the total CBL thickness and the maximum O_2 concentration at the coral surface. Values of slopes and fluxes are presented in µmol cm^{-2} h^{-1}. Acy, *Acropora cytherea*; Pve, *Pocillopora verrucosa*; Pcy, *Porites cylindrica*; OA, ocean acidification.

Species	Colony	Fragment	Flow	Light	Treatment	Slope[1]	Flux[1]	Slope[2]	Flux[2]
Acy	B	1	moderate	light	OA	-2,43	0,2	-5,26	0,43
Acy	B	1	low	light	OA	-2,91	0,24	-2,94	0,24
Acy	B	1	low	dark	OA	1,6	-0,13	1,41	-0,12
Acy	B	2	low	dark	OA	1,26	-0,1	1,34	-0,11
Acy	C	7	low	light	OA	-1,39	0,11	-2,12	0,17
Acy	C	7	low	dark	OA	1,04	-0,09	1,89	-0,16
Pve	B	5	moderate	light	control	-8,97	0,74	-6,16	0,51
Pve	C	7	low	light	OA	-1,14	0,09	-3,17	0,26
Pcy	A	2	low	light	OA	-2,42	0,2	-1,35	0,11
Pcy	A	2	low	dark	OA	0,99	-0,08	0,94	-0,08
Pcy	A	5	low	light	control	-0,82	0,07	-4,69	0,39
Pcy	A	5	low	dark	control	1,02	-0,08	2,08	-0,17
Pcy	B	1	low	light	OA	-1,92	0,16	-5,33	0,44
Pcy	B	1	low	dark	OA	0,37	-0,03	1,4	-0,12
Pcy	B	7	low	light	OA	-0,7	0,06	-1,51	0,12
Pcy	B	7	low	dark	OA	0,44	-0,04	0,57	-0,05
Pcy	C	4	low	light	control	-3,8	0,31	-1,16	0,1
Pcy	C	4	low	dark	control	0,38	-0,03	0,83	-0,07
Pcy	C	5	low	dark	control	0,74	-0,06	2,01	-0,17
Pcy	C	6	low	light	control	-3,56	0,29	-2,61	0,22
Pcy	C	6	moderate	dark	control	1,87	-0,15	3,15	-0,26
Pcy	C	6	low	dark	control	1,3	-0,11	0,98	-0,08
Pcy	C	7	low	dark	OA	0,69	-0,06	0,78	-0,06

References

1. Pacherres, C. O., Ahmerkamp, S., Schmidt-Grieb, G. M., Holtappels, M. & Richter, C. Ciliary vortex flows and oxygen dynamics in the coral boundary layer. *Sci Rep* **10**, 7541 (2020).

2. Martins, C. P. P., Wall, M., Schubert, P., Wilke, T. & Ziegler, M. Variability of the surface boundary layer of reef-building coral species. *Preprint (Version 1) at Research Square* (2024) doi:10.21203/rs.3.rs-3860900/v1.

3. Shapiro, O. H. *et al.* Vortical ciliary flows actively enhance mass transport in reef corals. *Proceedings of the National Academy of Sciences* **111**, 13391–13396 (2014).

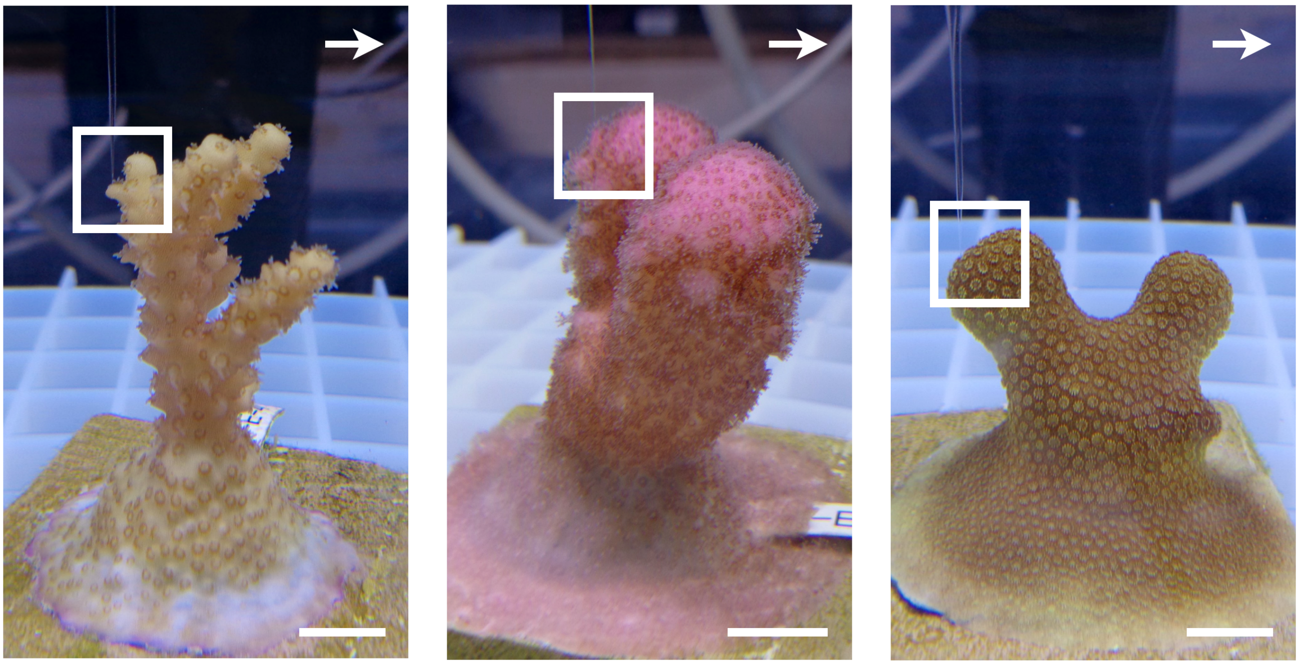

Supplementary Fig. S1. Photographs of *Acropora cytherea*, *Pocillopora verrucosa*, and *Porites cylindrica* (from left to right) during microsensor measurements. Rectangles mark representative locations of microsensor measurements across coral fragments and arrows indicate the direction of water flow. Scale bar = 1 cm approx.

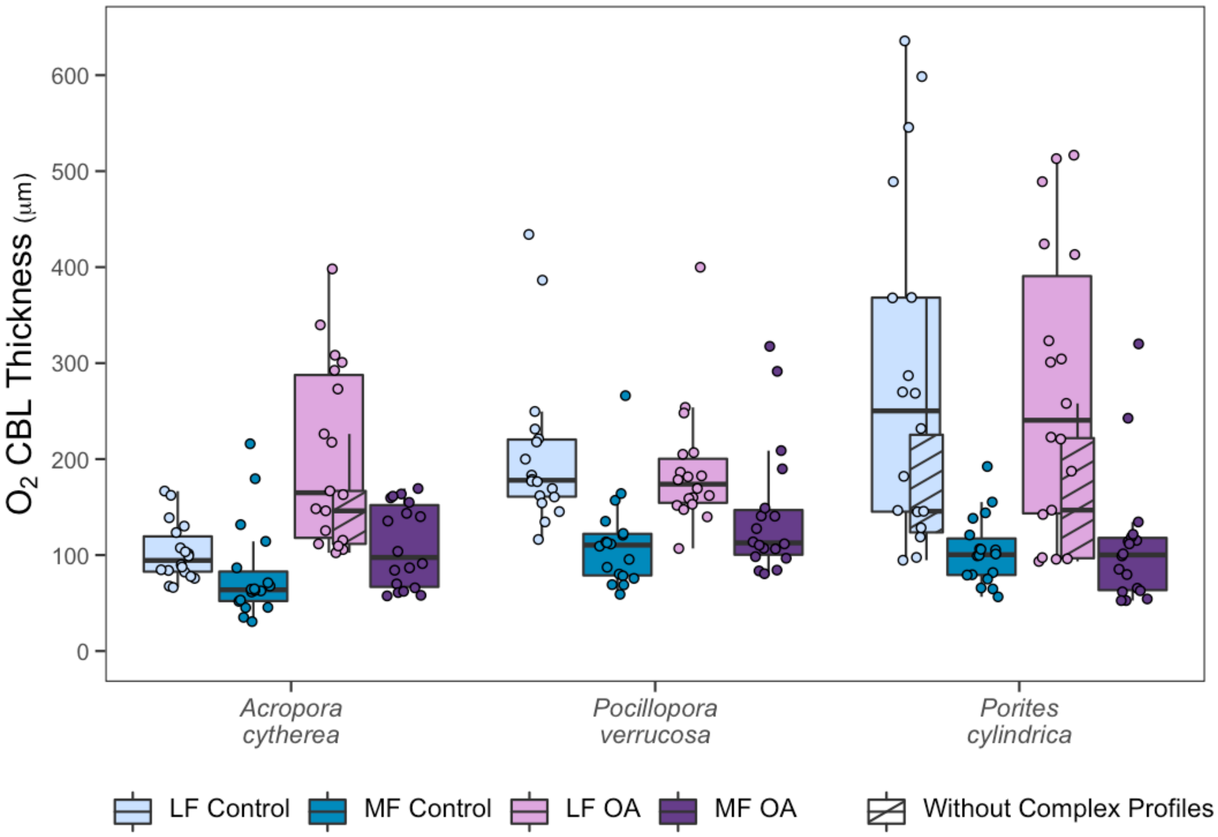

Supplementary Fig. S2. Visualisation of the effects of water flow and ocean acidification (OA) on the thickness of the concentration boundary layer (CBL) of *Acropora cytherea*, *Pocillopora verrucosa*, and *Porites cylindrica*, with and without including complex profiles (i.e., microsensor profiles with multiple linear gradients within the CBL). Profiles were measured in light and dark combined with low flow (LF, 2 cm s^{-1}) and moderate flow (MF, 6 cm s^{-1}) and values are presented pooled over light conditions. No complex profiles were observed for the H^+ CBL. Boxes represent the first and third quartiles with lines as medians and whiskers as the minimum and maximum values or up to the 1.5 * interquartile range (IQR), whichever is reached first.

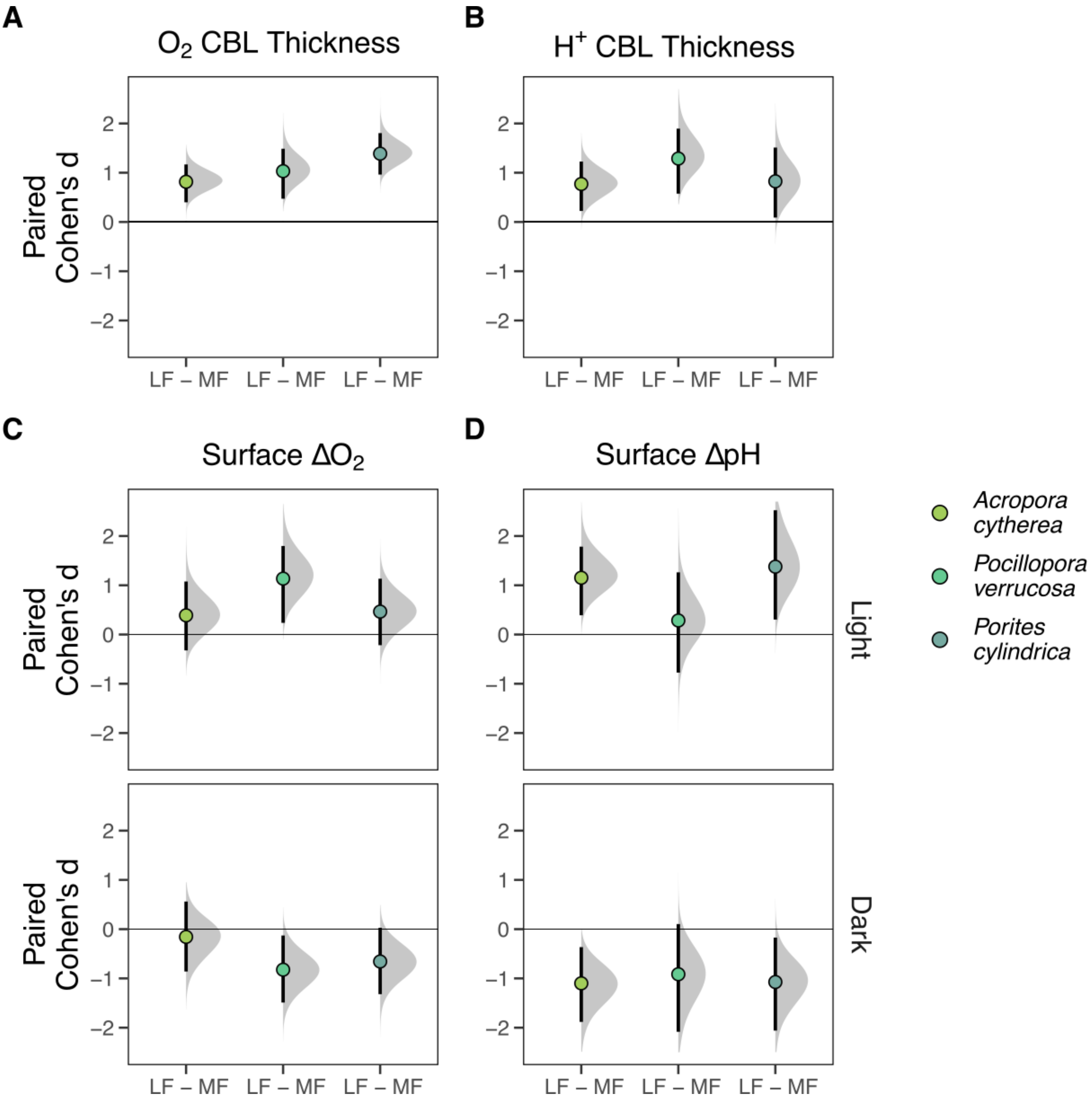

Supplementary Fig. S3. Visualisation of the effect size of flow effects on the concentration boundary layer (CBL) of *Acropora cytherea*, *Pocillopora verrucosa*, and *Porites cylindrica*. Cohen's d effect size between low flow (LF, 2 cm s^{-1}) and moderate flow (MF, 6 cm s^{-1}) for (A) O_2 CBL thickness, (B) H^+ CBL thickness, (C) O_2 concentration change at the coral surface relative to bulk seawater concentration (surface ΔO_2), and (D) pH change at the coral surface relative to bulk seawater level (surface ΔpH), measured in light and dark. Values of CBL thickness are pooled over light conditions. Black vertical lines are 95% confidence intervals from nonparametric bootstrap resampling and grey-shaded areas illustrate the resampled distribution of the effect size.

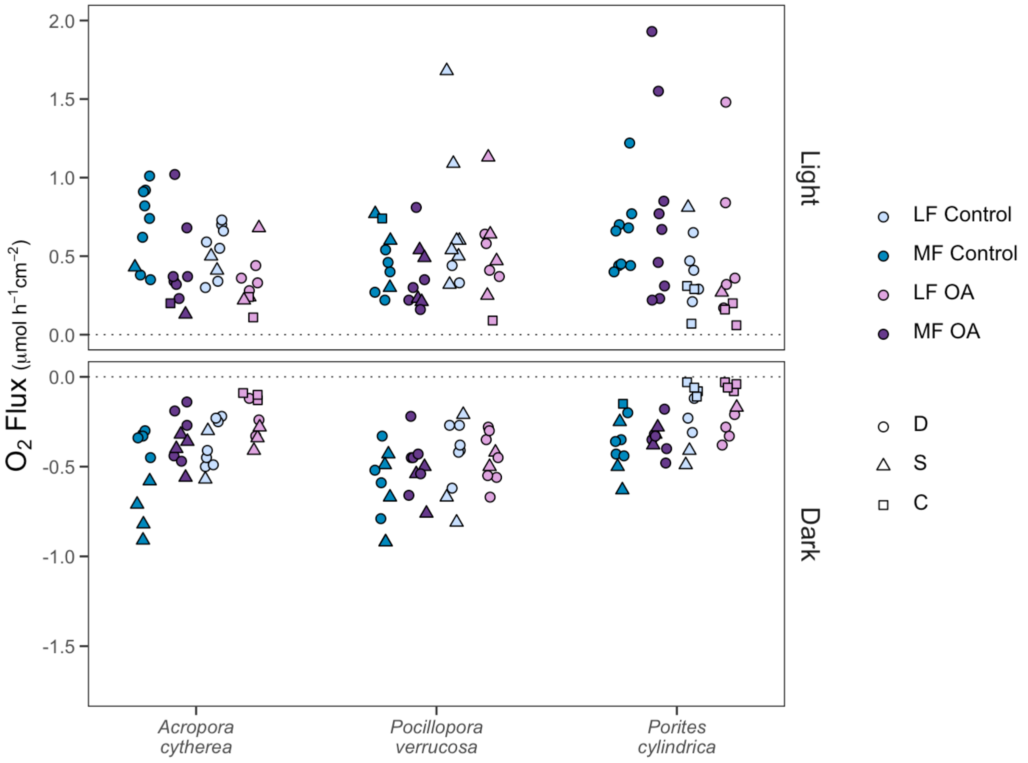

Supplementary Fig. S4. Visualisation of the effects of water flow and ocean acidification (OA) on the O_2 flux of *Acropora cytherea*, *Pocillopora verrucosa*, and *Porites cylindrica*, derived from O_2 concentration profiles categorised into three main profile shapes (D, diffusive; S, S-shaped; C, complex). Profiles were measured in light and dark combined with low flow (LF, 2 cm s^{-1}) and moderate flow (MF, 6 cm s^{-1}).

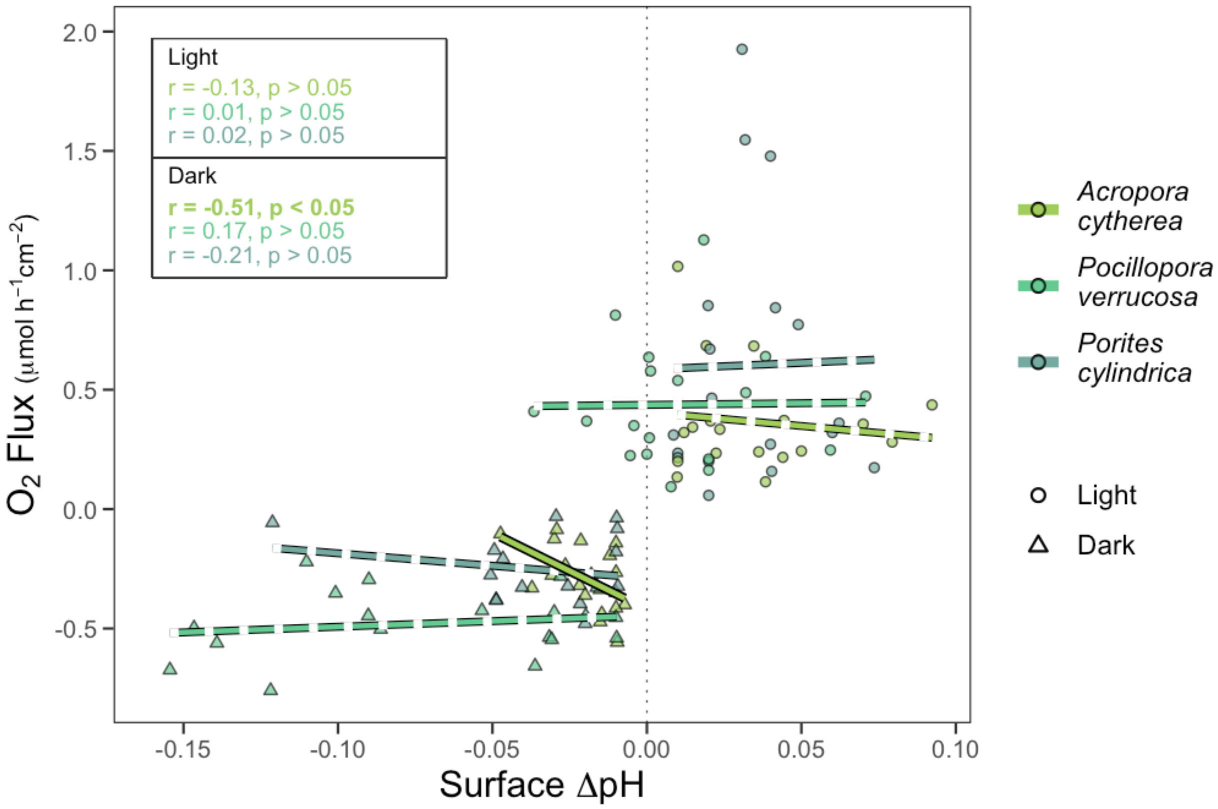

Supplementary Fig. S5. Relationship between pH changes at the coral surface relative to bulk seawater pH (surface ΔpH) and O_2 flux across the concentration boundary layer of *Acropora cytherea*, *Pocillopora verrucosa*, and *Porites cylindrica* in the ocean acidification treatment, measured in light and dark. Measurements under low and moderate flow are pooled together. Significant Pearson correlation coefficients (r) at the level of $\alpha < 0.05$ are marked in bold. Lines are linear regression fitting for significant (solid) and non-significant (dashed) correlations.

Supplementary Table S1. Details on coral species used in the experiment.

Species	Number of colonies	Origin	Collection year	CITES number
Acropora cytherea	3	Saudi Arabia (Red Sea)	2019	19-SA-000089-PD
Pocillopora verrucosa	2<br>1	Saudi Arabia (Red Sea)<br>Indonesia (Indo-Pacific)	2015<br>2007	15-SA-000882-PD<br>14846/IV/SATS-LN/2007
Porites cylindrica	3	Indonesia (Indo-Pacific)	2017	17nl241924/11

Supplementary Table S2. Brands of artificial-seawater salt used alternately during the acclimation and experimental period in the 'Ocean2100' experimental facility.

Salt	Brand
Deep Blue Sea-Salt	AquaPerfekt, Germany
Instant Ocean	Aquarium Systems, France
Reef Crystals	Aquarium Systems, France
Sea Salt	Aquaforest, Poland

Supplementary Table S3. Number of complex profiles measured in *Acropora cytherea*, *Pocillopora verrucosa*, and *Porites cylindrica* in the control and ocean acidification (OA) treatments and under low flow (2 cm s^{-1}) and moderate flow (6 cm s^{-1}).

Species	Flow	Treatment	*n*
Acropora cytherea	Low	Control	2
Acropora cytherea	Low	OA	5
Acropora cytherea	Moderate	Control	1
Acropora cytherea	Moderate	OA	1
Pocillopora verrucosa	Low	OA	1
Pocillopora verrucosa	Moderate	Control	1
Porites cylindrica	Low	Control	7
Porites cylindrica	Low	OA	8
Porites cylindrica	Moderate	Control	1
Porites cylindrica	Moderate	OA	1

Supplementary Table S4. Numerical output of linear mixed-effects models (LMMs) of concentration boundary layer (CBL) thickness of O_2 and H^+ gradients (pooled over light conditions). Global models were constructed with species (3 levels: *Acropora cytherea* [Acy], *Pocillopora verrucosa* [Pve], and *Porites cylindrica* [Pcy]) as a fixed factor. The global model of H^+ CBL thickness was computed using corals only from the ocean acidification (OA) treatment (see Materials and Methods). Models of O_2 CBL thickness of individual species and the model of H^+ CBL thickness of *A. cytherea* were constructed with flow (2 levels: low and moderate) and treatment (2 levels: control and OA) as fixed factors in a fully crossed design. Models of H^+ CBL thickness of *P. verrucosa* and *P. cylindrica* were constructed with the same structure, but without the treatment factor. All models were constructed with coral fragment identity (ID), colony, day of measurement (Day), and tank as random factors, except when the factor had near-zero variance, and treatment was additionally incorporated into global models as a random factor following the same guideline. Model formulas are specified. Log-transformation was applied to O_2 CBL thickness (global model) to meet model assumptions. σ^2, residual variance; τ_{00}, random intercept variance; ICC, intra-class correlation coefficient; N, number of levels of random effects groups; Marginal R^2, variance explained by the fixed effects; Conditional R^2, variance explained by the entire model

		O_2 CBL Thickness		H^+ CBL Thickness	
All Species		~ Species + (1 | ID) + (1 | Tank) + (1 | Treatment)		~ Species + (1 | ID) + (1 | Colony)	
	Fixed Effects	Estimates	SE	Estimates	SE
	(Intercept)	2.027	0.051	106.76	18.51
	species [Pve]	0.143	0.057	-0.38	26.17
	species [Pcy]	0.151	0.057	-3.47	26.17
	Random Effects				
	σ^2	0.038		2238.17	
	τ_{00}	0.020 ID		1446.18 ID	
		0.001 Tank		478.62 Colony	
		0.002 Treatment			
	ICC	0.37		0.41	
	N	54 ID		27 ID	
		6 Tank		9 Colony	
		2 Treatment			
	Observations	216		108	
	Marginal R^2 / Conditional R^2	0.074 / 0.416		0.001 / 0.412	
Acropora cytherea		~ Treatment + Flow + Treatment:Flow + (1 | ID) + (1| Day)		~ Treatment + Flow + Treatment:Flow + (1 | ID) + (1| Colony) + (1| Tank)	
	Fixed Effects	Estimates	SE	Estimates	SE
	(Intercept)	104.68	26.78	110.40	15.60
	treatment [OA]	96.14	24.37	18.08	20.70
	flow [moderate]	-22.41	11.16	-33.53	15.02
	treatment [OA] * flow [moderate]	-71.05	15.78	-9.92	20.65
	Random Effects				
	σ^2	1120.07		1806.00	
	τ_{00}	2090.31 ID		709.08 ID	
		843.07 Day		51.92 Colony	
				70.18 Tank	
	ICC	0.72		0.32	
	N	18 ID		17 ID	
		2 Day		3 Colony	
				6 Tank	
	Observations	72		68	
	Marginal R^2 / Conditional R^2	0.342 / 0.818		0.141 / 0.411	

Pocillopora verrucosa		~ Treatment + Flow + Treatment:Flow + (1 | ID)		~ Flow + (1 | ID) + (1| Colony) + (1| Tank)	
	Fixed Effects	Estimates	SE	Estimates	SE
	(Intercept)	205.45	21.01	146.17	27.54
	treatment [OA]	-17.02	29.71		
	flow [moderate]	-92.82	12.48	-79.59	13.27
	treatment [OA] * flow [moderate]	46.51	17.64		
	Random Effects				
	σ^2	1400.66		1584.29	
	τ_{00}	3271.42 $_{ID}$		843.70 $_{ID}$	
				1258.89 $_{Colony}$	
				471.38 $_{Tank}$	
	ICC	0.70		0.62	
	N	18 $_{ID}$		9 $_{ID}$	
				3 $_{Colony}$	
				3 $_{Tank}$	
	Observations	72		36	
	Marginal R^2 / Conditional R^2	0.227 / 0.768		0.281 / 0.726	
Porites cylindrica		~ Treatment + Flow + Treatment:Flow + (1 | ID)) + (1| Day)		~ Flow + (1 | ID) + (1| Colony)	
	Fixed Effects	Estimates	SE	Estimates	SE
	(Intercept)	286.84	37.82	123.06	18.71
	treatment [OA]	-22.30	50.80		
	flow [moderate]	-180.57	29.67	-39.54	8.59
	treatment [OA] * flow [moderate]	22.13	41.96		
	Random Effects				
	σ^2	7921.87		664.66	
	τ_{00}	7237.68 $_{ID}$		1357.44 $_{ID}$	
		526.87 $_{Day}$		486.79 $_{Colony}$	
	ICC	0.49		0.74	
	N	18 $_{ID}$		9 $_{ID}$	
		3 $_{Day}$		3 $_{Colony}$	
	Observations	72		36	
	Marginal R^2 / Conditional R^2	0.319 / 0.656		0.138 / 0.772	

Supplementary Table S5. Numerical output of linear mixed-effects models (LMMs) of change in O_2 concentration (surface ΔO_2) and pH (surface ΔpH) at the coral surface relative to bulk seawater, and of O_2 flux across the concentration boundary layer, measured in light and dark. Global models were constructed with species (3 levels: *Acropora cytherea* [Acy], *Pocillopora verrucosa* [Pve], and *Porites cylindrica* [Pcy]) as a fixed factor. Global models of surface ΔpH were computed using corals only from the ocean acidification (OA) treatment (see Materials and Methods). Models of surface ΔO_2 and O_2 flux of individual species and the model of surface ΔpH of *A. cytherea* were constructed with flow (2 levels: low and moderate) and treatment (2 levels: control and OA) as fixed factors in a fully crossed design. Models of surface ΔpH of *P. verrucosa* and *P. cylindrica* were constructed with the same structure, but without the treatment factor. All models were constructed with coral fragment identity (ID), colony, day of measurement (Day), and tank as random factors, except when the factor had near-zero variance, and treatment was additionally incorporated into global models as a random factor following the same guideline. Model formulas are specified. Square-root transformation was applied to dark surface ΔpH of *P. cylindrica* to meet model assumptions. σ^2, residual variance; τ_{00}, random intercept variance; ICC, intra-class correlation coefficient; N, number of levels of random effects groups; Marginal R^2, variance explained by the fixed effects; Conditional R^2, variance explained by the entire model

<table>
<tr><td></td><td></td><td colspan="2">Surface ΔO_2</td><td colspan="2">Surface ΔpH</td><td colspan="2">O_2 Flux</td></tr>
<tr><td>All Species</td><td></td><td colspan="2">~ species + (1 | ID) + (1 | Day) + (1 | Tank)</td><td colspan="2">~ species + (1 | ID)</td><td colspan="2">~ species + (1 | ID) + (1 | Tank) + (1 | Treatment)</td></tr>
<tr><td></td><td>Fixed Effects</td><td>Estimates</td><td>SE</td><td>Estimates</td><td>SE</td><td>Estimates</td><td>SE</td></tr>
<tr><td>Light</td><td>(Intercept)</td><td>67.85</td><td>12.18</td><td>0.035</td><td>0.006</td><td>-0.375</td><td>0.081</td></tr>
<tr><td></td><td>species [Pve]</td><td>6.50</td><td>16.87</td><td>-0.023</td><td>0.009</td><td>0.011</td><td>0.079</td></tr>
<tr><td></td><td>species [Pcy]</td><td>20.77</td><td>16.29</td><td>-0.002</td><td>0.009</td><td>0.005</td><td>0.079</td></tr>
<tr><td></td><td colspan="7">Random Effects</td></tr>
<tr><td></td><td>σ^2</td><td colspan="2">620.84</td><td colspan="2">< 0.001</td><td colspan="2">0.055</td></tr>
<tr><td></td><td>τ_{00}</td><td colspan="2">635.52 $_{ID}$</td><td colspan="2">< 0.001 $_{ID}$</td><td colspan="2">0.015 $_{ID}$</td></tr>
<tr><td></td><td></td><td colspan="2">191.51 $_{Day}$</td><td colspan="2"></td><td colspan="2">0.007 $_{Tank}$</td></tr>
<tr><td></td><td></td><td colspan="2">0.51 $_{Tank}$</td><td colspan="2"></td><td colspan="2">0.006 $_{Treatment}$</td></tr>
<tr><td></td><td>ICC</td><td colspan="2">0.57</td><td colspan="2">0.24</td><td colspan="2">0.34</td></tr>
<tr><td></td><td>N</td><td colspan="2">54 $_{ID}$</td><td colspan="2">27 $_{ID}$</td><td colspan="2">54 $_{ID}$</td></tr>
<tr><td></td><td></td><td colspan="2">8 $_{Day}$</td><td colspan="2"></td><td colspan="2">6 $_{Tank}$</td></tr>
<tr><td></td><td></td><td colspan="2">6 $_{Tank}$</td><td colspan="2"></td><td colspan="2">2 $_{Treatment}$</td></tr>
<tr><td></td><td>Observations</td><td colspan="2">108</td><td colspan="2">54</td><td colspan="2">108</td></tr>
<tr><td></td><td>Marginal R^2 / Conditional R^2</td><td colspan="2">0.050 / 0.593</td><td colspan="2">0.173 / 0.374</td><td colspan="2">0.000 / 0.339</td></tr>
<tr><td></td><td colspan="7"></td></tr>
<tr><td>Dark</td><td></td><td colspan="2">~ species + (1 | ID) + (1 | Day)</td><td colspan="2">~ species + (1 | ID)</td><td colspan="2">~ species + (1 | ID) + (1 | Tank) + (1 | Treatment)</td></tr>
<tr><td></td><td>(Intercept)</td><td>-44.41</td><td>5.75</td><td>-0.020</td><td>0.009</td><td>-0.372</td><td>0.050</td></tr>
<tr><td></td><td>species [Pve]</td><td>-33.84</td><td>8.00</td><td>-0.051</td><td>0.013</td><td>-0.138</td><td>0.046</td></tr>
<tr><td></td><td>species [Pcy]</td><td>-1.54</td><td>7.73</td><td>-0.012</td><td>0.013</td><td>0.101</td><td>0.046</td></tr>
<tr><td></td><td colspan="7">Random Effects</td></tr>
<tr><td></td><td>σ^2</td><td colspan="2">174.53</td><td colspan="2">0.001</td><td colspan="2">0.022</td></tr>
</table>

		τ_{00}	156.00 ID		< 0.001 ID		0.007 ID	
			39.03 Day				< 0.001 Tank	
							0.003 Treatment	
		ICC	0.53		0.33		0.29	
		N	54 ID		27 ID		54 ID	
			8 Day				6 Tank	
							2 Treatment	
		Observations	108		54		108	
		Marginal R^2 / Conditional R^2	0.385 / 0.709		0.301 / 0.532		0.226 / 0.475	
Acropora cytherea			~ Treatment + Flow + Treatment:Flow + (1 | ID) + (1 | Day)		~ Treatment + Flow + Treatment:Flow + (1 | ID)		~ Treatment + Flow + Treatment:Flow + (1 | ID)	
		Fixed Effects	Estimates	SE	Estimates	SE	Estimates	SE
	Light	(Intercept)	75.68	19.33	0.048	0.007	0.533	0.073
		treatment [OA]	-4.16	19.45	0.002	0.010	-0.210	0.103
		flow [moderate]	-6.58	5.17	-0.017	0.006	0.138	0.063
		treatment [OA] * flow [moderate]	-9.85	7.32	-0.014	0.008	-0.053	0.089
		Random Effects						
		σ^2	120.45		< 0.001		0.018	
		τ_{00}	366.03 ID		< 0.001 ID		0.030 ID	
			638.69 Day					
		ICC	0.89		0.66		0.63	
		N	18 ID		17 ID		18 ID	
			2 Day					
		Observations	36		34		36	
		Marginal R^2 / Conditional R^2	0.052 / 0.898		0.291 / 0.758		0.271 / 0.729	
	Dark		~ Treatment + Flow + Treatment:Flow + (1 | ID) + (1 | Day)		~ Treatment + Flow + Treatment:Flow + (1 | ID)		~ Treatment + Flow + Treatment:Flow + (1 | ID)	
		(Intercept)	-46.63	10.23	-0.024	0.003	-0.382	0.054
		treatment [OA]	0.39	3.18	-0.004	0.005	0.155	0.076
		flow [moderate]	0.39	3.18	0.006	0.004	-0.148	0.050
		treatment [OA] * flow [moderate]	5.18	4.50	0.008	0.006	0.024	0.071
		Random Effects						
		σ^2	45.51		< 0.001		0.011	
		τ_{00}	225.05 ID		< 0.001 ID		0.015 ID	
			148.84 Day					
		ICC	0.89		0.12		0.57	
		N	18 ID		17 ID		18 ID	

		2 Day					
	Observations	36		34		36	
	Marginal R^2 / Conditional R^2	0.019 / 0.894		0.266 / 0.355		0.313 / 0.703	
Pocillopora verrucosa		~ Treatment + Flow + Treatment:Flow + (1 | ID) + (1 | Colony)		~ Flow + (1 | ID)		~ Treatment + Flow + Treatment:Flow + (1 | ID)	
	Fixed Effects	Estimates	SE	Estimates	SE	Estimates	SE
Light	(Intercept)	94.71	13.89	0.016	0.0090	0.677	0.101
	treatment [OA]	7.18	17.37			-0.169	0.142
	flow [moderate]	-36.03	9.71	-0.009	0.0084	-0.201	0.132
	treatment [OA] * flow [moderate]	-13.26	13.73			0.061	0.186
	Random Effects						
	σ^2	424.46		< 0.001		0.078	
	τ_{00}	933.95 ID		< 0.001 ID		0.013 ID	
		125.61 Colony					
	ICC	0.71		0.57		0.15	
	N	18 ID		9 ID		18 ID	
		3 Colony					
	Observations	36		18		36	
	Marginal R^2 / Conditional R^2	0.244 / 0.784		0.026 / 0.577		0.122 / 0.249	
Dark		~ Treatment + Flow + Treatment:Flow + (1 | ID) + (1 | Colony) + (1 | Tank)		~ Flow + (1 | ID)		~ Treatment + Flow + Treatment:Flow + (1 | ID)	
	(Intercept)	-87.54	9.29	-0.093	0.015	-0.450	0.060
	treatment [OA]	1.32	12.61			-0.003	0.084
	flow [moderate]	15.01	5.73	0.042	0.013	-0.180	0.040
	treatment [OA] * flow [moderate]	4.13	8.10			0.127	0.057
	Random Effects						
	σ^2	147.73		< 0.001		0.007	
	τ_{00}	196.33 ID		0.001 ID		0.024 ID	
		20.32 Colony					
		123.91 Tank					
	ICC	0.70		0.62		0.77	
	N	18 ID		9 ID		18 ID	
		3 Colony					
		6 Tank					
	Observations	36		18		36	
	Marginal R^2 / Conditional R^2	0.139 / 0.740		0.181 / 0.689		0.147 / 0.804	

Porites cylindrica		~ Treatment + Flow + Treatment:Flow + (1 | ID) + (1 | Day) + (1 | Tank)		~ Flow + (1 | ID)		~ Treatment + Flow + Treatment:Flow + (1 | ID) + (1 | Tank)	
	Fixed Effects	Estimates	SE	Estimates	SE	Estimates	SE
Light	(Intercept)	97.41	17.65	0.044	0.005	0.391	0.178
	treatment [OA]	1.10	24.19			0.038	0.252
	flow [moderate]	-17.83	7.36	-0.022	0.004	0.257	0.140
	treatment [OA] * flow [moderate]	0.87	10.41			0.091	0.198
	Random Effects						
	σ^2	243.76		< 0.001		0.088	
	τ_{00}	829.42 $_{ID}$		< 0.001 $_{ID}$		0.047 $_{ID}$	
		71.16 $_{Day}$				0.050 $_{Tank}$	
		501.47 $_{Tank}$					
	ICC	0.85		0.65		0.52	
	N	18 $_{ID}$		9 $_{ID}$		18 $_{ID}$	
		3 $_{Day}$				6 $_{Tank}$	
		6 $_{Tank}$					
	Observations	36		18		36	
	Marginal R^2 / Conditional R^2	0.046 / 0.859		0.333 / 0.768		0.122 / 0.581	
Dark		~ Treatment + Flow + Treatment:Flow + (1 | ID) + (1 | Colony) + (1 | Tank)		~ Flow + (1 | ID)		~ Treatment + Flow + Treatment:Flow + (1 | ID)	
	(Intercept)	-57.16	5.46	0.200	0.020	-0.204	0.045
	treatment [OA]	13.13	7.30			0.030	0.064
	flow [moderate]	12.81	6.04	-0.064	0.014	-0.165	0.064
	treatment [OA] * flow [moderate]	-6.69	8.54			0.002	0.091
	Random Effects						
	σ^2	163.93		< 0.001		0.019	
	τ_{00}	0.79 $_{ID}$		0.003 $_{ID}$		< 0.001 $_{ID}$	
		9.46 $_{Colony}$					
		24.92 $_{Tank}$					
	ICC	0.14		0.74		< 0.01	
	N	18 $_{ID}$		9 $_{ID}$		18 $_{ID}$	
		3 $_{Colony}$					
		6 $_{Tank}$					
	Observations	36		18		36	
	Marginal R^2 / Conditional R^2	0.203 / 0.343		0.231 / 0.801		0.279 / 0.279	

Supplementary Table S6. Summary of the complete recording of temperature and pH during the experiment. Values are expressed as mean ± 1 SD with measurement replication (n). pH_T, pH on the total scale

Tank	Treatment Name	Temperature (ºC)	pH_T	Daily Minimum pH_T	Daily Maximum pH_T
1	Control	25.9 ± 0.2 (5,054)	7.94 ± 0.13 (4,927)	7.75 ± 0.06 (110)	8.11 ± 0.08 (110)
2	Ocean Acidification	25.9 ± 0.2 (4,573)	7.74 ± 0.14 (4,727)	7.56 ± 0.08 (110)	7.92 ± 0.09 (110)
3	Control	25.9 ± 0.2 (5,010)	7.96 ± 0.13 (4,915)	7.77 ± 0.06 (110)	8.14 ± 0.07 (110)
4	Ocean Acidification	26.0 ± 0.2 (4,364)	7.76 ± 0.14 (4,853)	7.58 ± 0.09 (110)	7.95 ± 0.10 (110)
5	Control	25.7 ± 0.3 (4,717)	7.96 ± 0.14 (4,831)	7.77 ± 0.09 (110)	8.14 ± 0.09 (110)
6	Ocean Acidification	26.0 ± 0.2 (4,323)	7.77 ± 0.14 (4,785)	7.59 ± 0.08 (110)	7.96 ± 0.10 (110)

Supplementary Table S7. Results of ANOVAs testing differences between treatments in seawater carbonate parameters, from linear mixed-effects models (LMMs). Significant effects at the level of $\alpha < 0.05$ are marked in bold. fCO_2, fugacity of CO_2; DIC, dissolved inorganic carbon; Ω_{ar}, aragonite saturation; Ω_{ca}, calcite saturation

Variable	Effect	df	F	p
fCO_2	Treatment	1	217.1	**< 0.001**
DIC	Treatment	1	108.8	**< 0.001**
CO_2	Treatment	1	205.1	**< 0.001**
HCO_3^-	Treatment	1	165.7	**< 0.001**
CO_3^{2-}	Treatment	1	201.1	**< 0.001**
Ω_{ar}	Treatment	1	203.7	**< 0.001**
Ω_{ca}	Treatment	1	205.2	**< 0.001**

Supplementary Table S8. Summary of seawater conditions in the flume during microsensor measurements. Partial pressure of CO_2 (pCO_2), fugacity of CO_2 (fCO_2), dissolved inorganic carbon (DIC), CO_2, HCO_3^-, CO_3^{2-}, aragonite saturation (Ω_{ar}), and calcite saturation (Ω_{ca}) were calculated using values of total alkalinity (TA) measured during the period of microsensor measurements and daily recording of pH, temperature, and salinity. Values are expressed as mean ± 1 SD, with sample size (n) of measured parameters (salinity, temperature, pH, TA) and calculated parameters (pCO_2, fCO_2, DIC, CO_2, HCO_3^-, CO_3^{2-}, Ω_{ar}, Ω_{ca}). pH_T, pH on the total scale

Parameter	Control	Ocean Acidification
Salinity	34.6 ± 0.3 (8)	34.6 ± 0.2 (11)
Temperature (ºC)	26.0 ± 0.1 (14)	26.0 ± 0.0 (20)
pH_T	8.02 ± 0.02 (14)	7.77 ± 0.03 (20)
TA (µmol kg^{-1})	2,184 ± 8 (2)	2,239 (1)
pCO_2 (µatm)	407 ± 30 (14)	820 ± 63 (20)
fCO_2 (µatm)	406 ± 30 (14)	817 ± 63 (20)
DIC (µmol kg^{-1})	1,904 ± 22 (14)	2,077 ± 13 (20)
CO_2 (µmol kg^{-1})	11 ± 1 (14)	23 ± 2 (20)
HCO_3^- (µmol kg^{-1})	1,695 ± 29 (14)	1,928 ± 19 (20)
CO_3^{2-} (µmol kg^{-1})	197 ± 9 (14)	127 ± 8 (20)
Ω_{ar}	3.16 ± 0.14 (14)	2.03 ± 0.12 (20)
Ω_{ca}	4.77 ± 0.20 (14)	3.07 ± 0.18 (20)
O_2 (µM)	238 ± 9 (108)	241 ± 6 (108)

Supplementary Table S9. Post hoc analysis of global linear mixed-effects models (LMMs) with significant species differences in LMM-ANOVAs and of LMMs of individual species with significant interaction terms in LMM-ANOVAs. Species contrasts of surface ΔpH are from global models computed using coral fragments only from the ocean acidification treatment (see Materials and Methods). Summarised output of LMMs and model information can be found in Supplementary Tables S3, S4. p-values were adjusted with Bonferroni correction and significant effects with $\alpha < 0.05$ are marked in bold. Acy, *Acropora cytherea*; Pve, *Pocillopora verrucosa*; Pcy, *Porites cylindrica*; CBL, concentration boundary layer; surface ΔO_2, change in surface O_2 concentration relative to bulk seawater concentration; surface ΔpH, change in surface pH relative to bulk seawater pH; LF, low flow; MF, moderate flow

	Model Term	Light	Parameter	Contrast	df	t	p
Global Models	Species	Pooled	O_2 CBL Thickness	Acy vs. Pve	46.0	-2.52	**0.046**
	Species	Pooled	O_2 CBL Thickness	Acy vs. Pcy	46.0	-2.66	**0.032**
	Species	Pooled	O_2 CBL Thickness	Pve vs. Pcy	46.0	0.14	1.000
	Species	Dark	Surface ΔO_2	Acy vs. Pve	3.7	4.03	0.055
	Species	Dark	Surface ΔO_2	Acy vs. Pcy	3.9	0.20	1.000
	Species	Dark	Surface ΔO_2	Pve vs. Pcy	4.5	4.03	**0.037**
	Species	Light	Surface ΔpH	Acy vs. Pve	24.0	2.69	**0.039**
	Species	Light	Surface ΔpH	Acy vs. Pcy	24.0	0.20	1.000
	Species	Light	Surface ΔpH	Pve vs. Pcy	24.0	-2.48	0.061
	Species	Dark	Surface ΔpH	Acy vs. Pve	24.0	2.69	**0.038**
	Species	Dark	Surface ΔpH	Acy vs. Pcy	24.0	-9.94	**< 0.001**
	Species	Dark	Surface ΔpH	Pve vs. Pcy	24.0	-12.63	**< 0.001**
	Species	Dark	O_2 Flux	Acy vs. Pve	46.0	3.03	**0.012**
	Species	Dark	O_2 Flux	Acy vs. Pcy	46.0	-2.22	0.094
	Species	Dark	O_2 Flux	Pve vs. Pcy	46.0	5.25	**< 0.001**
Acropora cytherea	Treatment:Flow	Pooled	O_2 CBL Thickness	Control LF vs. OA LF	18.8	-3.92	**0.002**
	Treatment:Flow	Pooled	O_2 CBL Thickness	Control MF vs. OA MF	18.8	-1.02	0.638

Supplementary Table S10. Summary of quantitative traits of the concentration boundary layer (CBL) of *Acropora cytherea*, *Pocillopora verrucosa*, and *Porites cylindrica* measured in light and darkness, calculated for low flow (2 cm s^{-1}) and moderate flow (6 cm s^{-1}) conditions. Values are mean ± 1 SD with sample size (n) and are pooled over the control and ocean acidification (OA) treatments. CBL thickness values are additionally pooled over light conditions. Values of H^+ CBL thickness and surface ΔpH of *P. verrucosa* and *P. cylindrica* are from the OA treatment only (see Materials and Methods). surface ΔO_2, change in surface O_2 concentration relative to bulk seawater concentration; surface ΔpH, change in surface pH relative to bulk seawater pH

Species	Flow	Light	O_2 CBL Thickness (μm)	H^+ CBL Thickness (μm)	Surface ΔO_2 (μM)	Surface ΔpH	O_2 Flux (μmol cm^{-2} h^{-1})
Acropora cytherea	Low	Light	153 ± 86 (36)	120 ± 55 (34)	73.6 ± 24.8 (18)	0.05 ± 0.02 (17)	0.43 ± 0.19 (18)
	Low	Dark			-45.9 ± 15.0 (18)	-0.03 ± 0.01 (17)	-0.30 ± 0.15 (18)
	Moderate	Light	95 ± 48 (36)	81 ± 45 (34)	62.1 ± 31.6 (18)	0.03 ± 0.01 (17)	0.54 ± 0.29 (18)
	Moderate	Dark			-42.9 ± 21.2 (18)	-0.02 ± 0.01 (17)	-0.44 ± 0.21 (18)
Pocillopora verrucosa	Low	Light	197 ± 73 (36)	146 ± 78 (18)	98.3 ± 40.3 (18)	0.02 ± 0.04 (9)	0.59 ± 0.37 (18)
	Low	Dark			-86.9 ± 21.4 (18)	-0.09 ± 0.05 (9)	-0.45 ± 0.17 (18)
	Moderate	Light	127 ± 60 (36)	67 ± 34 (18)	55.6 ± 33.6 (18)	0.01 ± 0.01 (9)	0.42 ± 0.21 (18)
	Moderate	Dark			-69.8 ± 20.0 (18)	-0.05 ± 0.04 (9)	-0.57 ± 0.19 (18)
Porites cylindrica	Low	Light	277 ± 162 (36)	123 ± 53 (18)	98.3 ± 41.6 (18)	0.04 ± 0.02 (9)	0.41 ± 0.35 (18)
	Low	Dark			-50.6 ± 15.1 (18)	-0.05 ± 0.03 (9)	-0.19 ± 0.15 (18)
	Moderate	Light	107 ± 54 (36)	84 ± 42 (18)	81.0 ± 32.8 (18)	0.02 ± 0.01 (9)	0.71 ± 0.46 (18)
	Moderate	Dark			-41.1 ± 13.7 (18)	-0.02 ± 0.01 (9)	-0.35 ± 0.12 (18)

Supplementary Table S11. Summary of quantitative traits of the concentration boundary layer (CBL) of *Acropora cytherea*, *Pocillopora verrucosa*, and *Porites cylindrica* measured in light and darkness, calculated for the control and ocean acidification (OA) treatment. Values are mean ± 1 SD with sample size (n) and are pooled over water flow conditions. CBL thickness values are additionally pooled over light conditions. Surface ΔO_2, change in surface O_2 concentration relative to bulk seawater concentration; surface ΔpH, change in surface pH relative to bulk seawater pH

Species	Treatment	Light	O_2 CBL Thickness (µm)	H^+ CBL Thickness (µm)	Surface ΔO_2 (µM)	Surface ΔpH	O_2 Flux (µmol cm^{-2} h^{-1})
Acropora cytherea	Control	Light	91 ± 42 (36)	94 ± 53 (32)	70.5 ± 29.5 (18)	0.04 ± 0.02 (16)	0.60 ± 0.22 (18)
	Control	Dark			-45.6 ± 20.6 (18)	-0.02 ± 0.01 (16)	-0.46 ± 0.20 (18)
	OA	Light	156 ± 86 (36)	107 ± 54 (36)	65.2 ± 28.2 (18)	0.04 ± 0.02 (18)	0.37 ± 0.22 (18)
	OA	Dark			-43.3 ± 15.7 (18)	-0.02 ± 0.01 (18)	-0.29 ± 0.14 (18)
Pocillopora verrucosa	Control	Light	159 ± 82 (36)	n/a	76.7 ± 45.5 (18)	n/a	0.58 ± 0.35 (18)
	Control	Dark			-80.0 ± 22.1 (18)	n/a	-0.54 ± 0.22 (18)
	OA	Light	165 ± 69 (36)	106 ± 72 (36)	77.2 ± 40.7 (18)	0.01 ± 0.03 (18)	0.44 ± 0.26 (18)
	OA	Dark			-76.6 ± 22.8 (18)	-0.07 ± 0.05 (18)	-0.48 ± 0.14 (18)
Porites cylindrica	Control	Light	194 ± 156 (36)	n/a	87.7 ± 29.7 (18)	n/a	0.52 ± 0.28 (18)
	Control	Dark			-50.8 ± 16.3 (18)	n/a	-0.29 ± 0.17 (18)
	OA	Light	190 ± 140 (36)	103 ± 51 (36)	91.6 ± 45.6 (18)	0.03 ± 0.02 (18)	0.60 ± 0.54 (18)
	OA	Dark			-41.0 ± 12.0 (18)	-0.03 ± 0.03 (18)	-0.26 ± 0.14 (18)

Supplementary Table S12. Cohen's d effect size for ocean acidification (OA) effects on the O_2 CBL thickness of *Acropora cytherea* under low flow (LF) and moderate flow (MF), calculated for the dataset with and without the inclusion of complex profiles.

Dataset	Group 1	Group 2	Cohen's d	95 % Confidence Interval	
				Lower	Upper
With complex profiles	Control LF	OA LF	1.432	0.877	2.046
With complex profiles	Control MF	OA MF	0.624	-0.155	1.426
Without complex profiles	Control LF	OA LF	1.131	0.380	1.765
Without complex profiles	Control MF	OA MF	0.500	-0.262	1.328

Supplementary Table S13. Summary of change in O_2 concentration (surface ΔO_2) and pH (surface ΔpH) at the coral surface relative to bulk seawater levels, and O_2 flux across the concentration boundary layer of *Acropora cytherea*, *Pocillopora verrucosa*, and *Porites cylindrica* in light and darkness. Values are mean ± 1 SD with sample size (n) and are pooled over flow conditions and treatments, except for surface ΔpH.

Species	Treatment	Light	Surface ΔO_2 (µM)	Surface ΔpH	O_2 Flux (µmol cm^{-2} h^{-1})
Acropora cytherea	Control	Light	67.8 ± 28.6 (36)	0.04 ± 0.02 (16)	0.48 ± 0.25 (36)
	OA	Light		0.04 ± 0.02 (18)	
	Control	Dark	-44.4 ± 18.1 (36)	-0.02 ± 0.01 (16)	-0.37 ± 0.19 (36)
	OA	Dark		-0.02 ± 0.01 (18)	
Pocillopora verrucosa	Control	Light	77.0 ± 42.5 (36)	n/a	0.51 ± 0.31 (36)
	OA	Light		0.01 ± 0.03 (18)	
	Control	Dark	-78.3 ± 22.2 (36)	n/a	-0.51 ± 0.19 (36)
	OA	Dark		-0.07 ± 0.05 (18)	
Porites cylindrica	Control	Light	89.7 ± 38.0 (36)	n/a	0.56 ± 0.43 (36)
	OA	Light		0.03 ± 0.02 (18)	
	Control	Dark	-45.9 ± 15.0 (36)	n/a	-0.27 ± 0.16 (36)
	OA	Dark		-0.03 ± 0.03 (18)	
